# Drug Repurposing for Japanese Encephalitis Virus Infection by Systems Biology Methods

**DOI:** 10.3390/molecules23123346

**Published:** 2018-12-18

**Authors:** Bo-Min Lv, Xin-Yu Tong, Yuan Quan, Meng-Yuan Liu, Qing-Ye Zhang, Yun-Feng Song, Hong-Yu Zhang

**Affiliations:** 1 Hubei Key Laboratory of Agricultural Bioinformatics, College of Informatics, Huazhong Agricultural University, Wuhan 430070, China; lbm612@webmail.hzau.edu.cn (B.-M.L.); tongxinyu@webmail.hzau.edu.cn (X.-Y.T.); qyuan@webmail.hzau.edu.cn (Y.Q.); liumengyuan2017@outlook.com (M.-Y.L.); zqy@mail.hzau.edu.cn (Q.-Y.Z.); 2 State Key Laboratory of Agricultural Microbiology, Huazhong Agricultural University, Wuhan 430070, China; syf@mail.hzau.edu.cn

**Keywords:** Japanese encephalitis virus, drug repurposing, systems biology, antiviral agents

## Abstract

Japanese encephalitis is a zoonotic disease caused by the Japanese encephalitis virus (JEV). It is mainly epidemic in Asia with an estimated 69,000 cases occurring per year. However, no approved agents are available for the treatment of JEV infection, and existing vaccines cannot control various types of JEV strains. Drug repurposing is a new concept for finding new indication of existing drugs, and, recently, the concept has been used to discover new antiviral agents. Identifying host proteins involved in the progress of JEV infection and using these proteins as targets are the center of drug repurposing for JEV infection. In this study, based on the gene expression data of JEV infection and the phenome-wide association study (PheWAS) data, we identified 286 genes that participate in the progress of JEV infection using systems biology methods. The enrichment analysis of these genes suggested that the genes identified by our methods were predominantly related to viral infection pathways and immune response-related pathways. We found that bortezomib, which can target these genes, may have an effect on the treatment of JEV infection. Subsequently, we evaluated the antiviral activity of bortezomib using a JEV-infected mouse model. The results showed that bortezomib can lower JEV-induced lethality in mice, alleviate suffering in JEV-infected mice and reduce the damage in brains caused by JEV infection. This work provides an agent with new indication to treat JEV infection.

## 1. Introduction

The Japanese encephalitis virus (JEV) is the main pathogen that causes severe encephalitis in humans. JEV belongs to the genus of *Flavivirus*, which also includes other arboviruses, such as the Dengue virus (DENV), West Nile virus (WNV), and Zika virus (ZIKV) [1]. JEV is a positive-sense single-stranded RNA virus. The genome of JEV is approximately 11 kb in length, containing a single open reading frame (ORF) flanked by the 5′- and 3′-untranslated regions (UTRs). The ORF encodes a long polyprotein that is cleaved into three structural proteins (capsid [C], pre-membrane [prM], and envelope [E]) and seven nonstructural proteins (NS1, NS2A, NS2B, NS3, NS4A, NS4B, and NS5) [2]. The structural proteins make up the infectious viral particle and the nonstructural proteins participate in multiple steps of viral life cycle including viral replication, virion assembly, and immune evasion [2]. 

Since the first record of the virus in the late 1800s, JEV has posed a significant threat to global health [3]. It is reported that there are 69,000 cases of JEV infection per year [4]. The average mortality rate caused by JEV can be as high as 30% in the past 30 years, and the proportion of survival with permanent neurological or psychiatric sequelae is approximately 44% [1]. With its epidemic area expansion, JEV affects approximately 25 countries in Asia, and approximately 60% of the population lives with a risk of JEV infection [2]. At present, vaccination is the most effective way to prevent JEV infection. The common vaccines include the inactivated mouse brain-derived vaccine (JE-VAX), inactivated BHK-21 cell-derived vaccine, live-attenuated vaccine (SA14-14-2), inactivated Vero cell-derived vaccine, and the chimeric attenuated vaccine [5]. However, approximately 80% cases of the JEV infection occur in areas covered by the JEV vaccination program due to the failures of immunization strategies or the limitation of vaccines themselves [1]. To date, no clinically approved antiviral agents have been available for the treatment of JEV infection. Furthermore, few randomized clinical trials have tested treatments for JEV. In the past 30 years, only six agents for the treatment of JEV infection have been tested by clinical trials, but none of them have been found effective [1]. Therefore, it is essential and urgent to find a safe and effective treatment.

Drug repurposing has recently become a very popular method for drug discovery; drug repurposing provides old drugs (including approved drugs, under research drugs, and withdrawn drugs) with new indications by exploring new molecular pathways and targets [6,7]. With this strategy, finding an alternative agent to treatment JEV infection will be fast and safe. During the past decades, the traditional method for drug repurposing depends on high-throughput screening of small-molecule libraries consisting of approved and developing drugs [8]. However, the success rate of high-throughput screening for effective repurposed drugs has dropped dramatically [9]. With the development of computational methods, the high-throughput omics data, virtual screening, and text mining have been used for drug repurposing [9,10]. One of the computational methods for antiviral drug repurposing is to target pathogen to block its lifecycle. Using the crystal structure of the E protein and the strategy of structural-based virtual screening (SBVS), Leal et al. identified a compound exhibiting marked antiviral activity against DENV with its EC50 being 3.1 µM [11]. The other methods for antiviral drug repurposing are targeting host genes to inhibit pathogen infection. Identifying the proteins participating in the pathogen infection process is the basis of host-targeted drug repurposing approaches [9]. Quan et al. identified 170 *Mycobacterium tuberculosis* (Mtb) infection-associated genes by theoretical genetic analysis, and obtained high potential anti-Mtb drugs by targeting these genes [12]. Therefore, it is possible to rapidly identify effective therapeutics for JEV infection using the method of drug repurposing through targeting JEV-susceptible genes.

**Figure 1:**
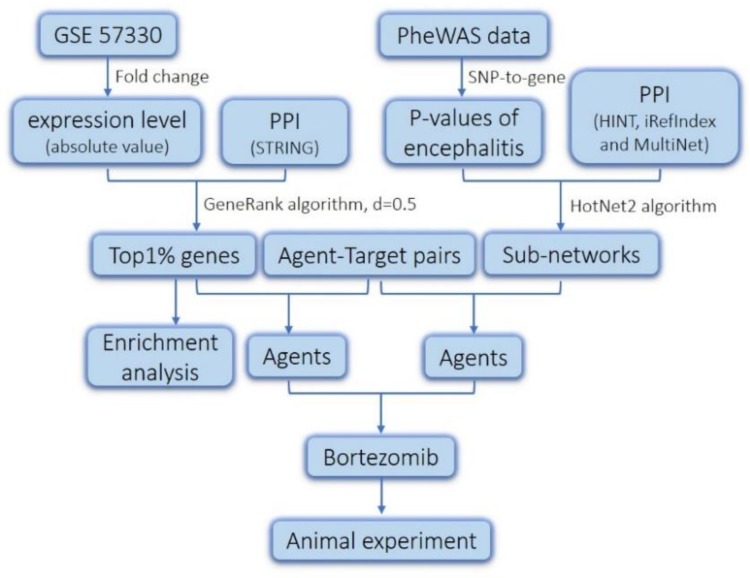
The pipeline for gene screening and drug repurposing. The dataset GSE57330 obtained from GEO database. The protein-protein interaction (PPI) network used in the HotNet2 algorithm was obtained from HINT, iRefIndex, and MultiNet. The protein-protein interaction (PPI) network used in the GeneRank algorithm was derived from the STRING database.

Systems biology has been used to identify the pathogenic mechanisms of complex human diseases by integrating genetic variation, genomics, pathways, and molecular networks [13]. The advent of systems biology provides a powerful method for facilitating drug development and drug repurposing [14]. The representative algorithms used in the systems biology field include GeneRank and HotNet2 [15,16]. In this study, we applied the methods of HotNet2 and GeneRank to identify the genes essential in JEV infection (Figure 1). Additionally, we analyzed Gene Ontology (GO) and Kyoto Encyclopedia of Genes and Genomes (KEGG) athway enrichment of these genes to validate our results. Using the information of the drug-target, we obtained the agents that have a potential treatment effect on JEV infection. We found that multiple targets of bortezomib play critical roles in the progress of JEV infection based on the analysis of the PheWAS data of encephalitis and of the gene expression data of human microglial cells after JEV infection. Furthermore, we investigated the effect of bortezomib using a JEV-infected mouse model. Overall, our research provided a novel agent for the treatment of JEV infection.

## 2. Results and Discussion

### 2.1. Screening of Genes Associated with JEV Infection by GeneRank Algorithm

The gene expression data could reveal the relationship between genes and JEV infection. Therefore, we resorted to the Gene Expression Omnibus (GEO)-contained gene expression datasets following JEV infection to identify the JEV-susceptible genes. The dataset GSE57330 includes 12 samples that were detected at three time points (6, 24, and 48 h) post JEV infection [17]. Taking the gene expression data detected at different time as a whole, we calculated the value of fold change using the mean-gene expression. Thus, we determined the genes that were upregulated and downregulated after JEV infection of human microglial cells. Ordinarily, the genes whose fold change values are at least two-fold above those of the uninfected group and that have a *p*-value < 0.05 are defined as significantly associated with JEV infection. However, this approach may ignore those genes associated with JEV infection, for which the expression was not significantly altered. Therefore, we used the GeneRank algorithm to identify genes associated with JEV infection.

The GeneRank algorithm was derived from the Google search engine PageRank [15]. It can take advantage of the biological network to identify key genes associated with diseases, regardless of whether their expression is altered significantly or not. To find the genes associated with JEV infection, we ranked genes with the GeneRank algorithm. Taking the absolute value of fold change as the initial importance of a gene, we obtained the order of functional genes participating in JEV infection. According to the result calculated by GeneRank, we defined the top 1% genes as significant genes involved in the JEV infection process (Table S1). As indicated in Table S1, several genes have been reported to affect the process of JEV infection. For example, the expression of the 2′,5′-oligoadenylate synthetases (OAS) family (OAS1, OAS2 and OASL) inhibited the replication of JEV in PK-15 cells in one previous study [18]. The members of the tripartite-motif containing (TRIM) protein were reported to be a negative regulator of IFN-β during JEV infection and to inhibit JEV replication by degrading the viral protein in some other studies [19,20]. The results suggested that the genes identified by the GeneRank algorithm may play critical roles in the lifecycle of JEV.

**Figure 2:**
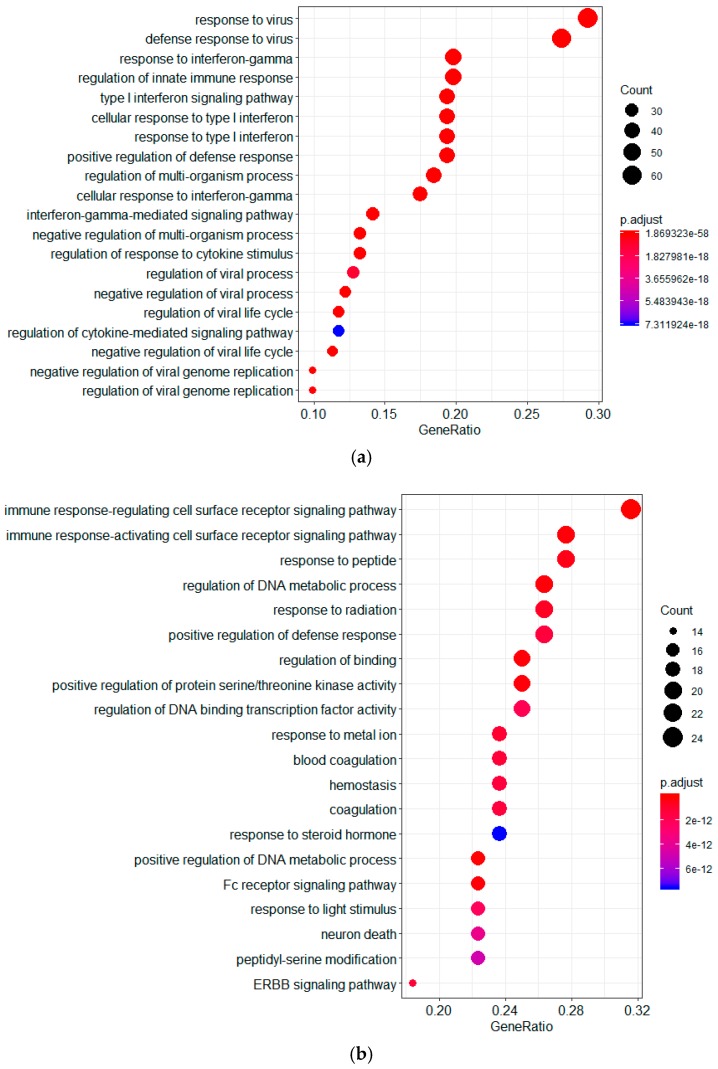

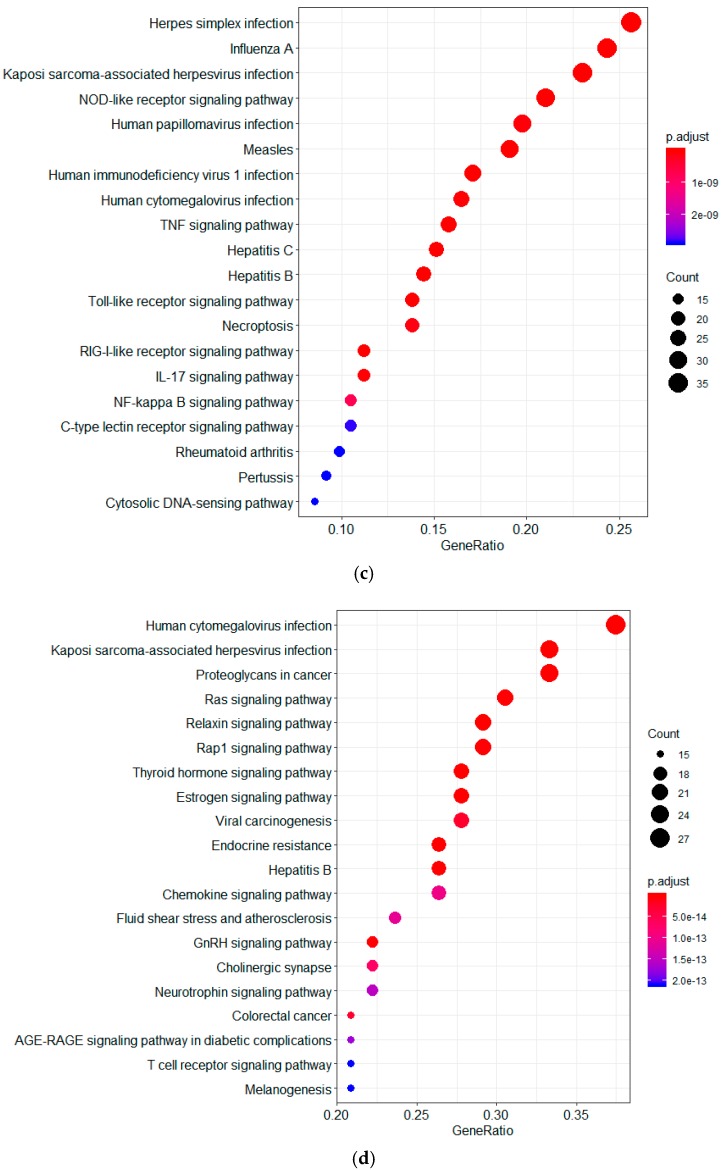
Functional characterization of the genes ranked by the GeneRank algorithm. Downregulated and upregulated genes that were ranked by the GeneRank algorithm were subjected to a GO enrichment analysis (biological processes) and a KEGG pathway enrichment analysis using the clusterProfiler package in R. The top 20 of the GO and pathways in that the up- and downregulated genes were significantly enriched, respectively (*p*. adjust-value < 1 × 10^−8^) are presented. (**a**) GO enrichment analysis of upregulated genes; (**b**) GO enrichment analysis of downregulated genes; (**c**) KEGG pathway enrichment analysis of upregulated genes; (**d**) KEGG pathway enrichment analysis of downregulated genes.

To understand the biological functional genes ranked by the GeneRank algorithm, a Gene Ontology (GO) enrichment analysis was conducted using the clusterProfiler package in R [21]. A *p*-value < 0.05 was used as the cutoff criterion. The results showed that these genes were involved in different cellular functions, including immune response, response to peptide, the regulation of DNA metabolic process, response to virus, response to interferon-γ, and the regulation of innate immune response (Figure 2). In addition, we investigated the involvement of these genes in signal transduction pathways using clusterProfiler package. As shown in Figure 2, the most significant KEGG pathways in which the downregulated genes were enriched included human cytomegalovirus infection, Kaposi sarcoma-associated herpesvirus infection, and proteoglycans in cancer. On the other hand, the upregulated genes were enriched in viral infection pathways (including herpes simplex infection, influenza A, Kaposi sarcoma-associated herpesvirus, and human papillomavirus infection) and NOD-like receptor signaling pathway. The results suggested that the genes ranked by the GeneRank algorithm were involved in viral infection pathways and immune response-related pathways.

### 2.2. Drug Repurposing for JEV Infection by Targeting GeneRank-Derived Genes

**Figure 3:**
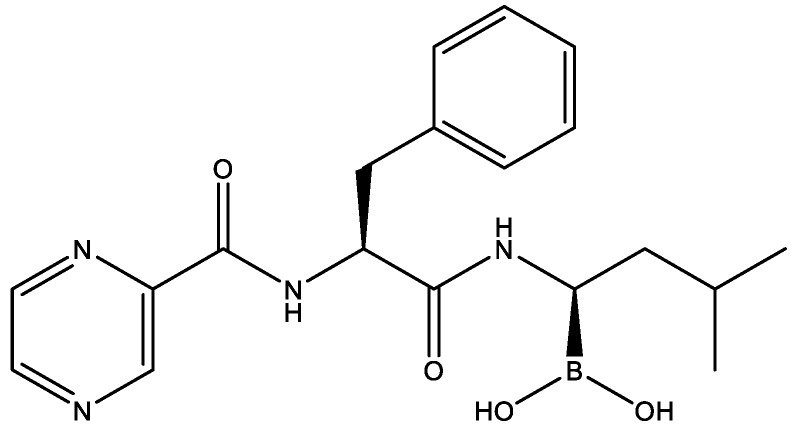
The chemical structure of bortezomib.

To identify approved drugs for the treatment of JEV infection, we collected the information about the association between chemical agents and its targets from the Drug-Gene Interaction database (DGIdb, http://dgidb.genome.wustl.edu/), the Therapeutic Target Database (TTD, http://bidd.nus.edu.sg/group/cjttd/) and the DrugBank (http://www.drugbank.ca/) [22,23,24]. By targeting the top 1% of genes derived from the GeneRank calculation, we obtained 91 agents that might have a potential effect on the treatment of JEV infection (Table S2). It should be noted that among these agents, we found bortezomib, which was reported to have the ability to inhibit DENV and ZIKV infection, with its chemical structure shown in Figure 3 [25,26]. Given that DENV, ZIKV, and JEV all belong to the genus of *flavivirus*, we speculated that bortezomib may have the potential ability to treat JEV infection. In addition to bortezomib, other agents, such as aspirin, curcumin, etanercept, and minocycline, were also found to have effects on the inhibition of JEV infection (Table 1) [27,28,29,30,31,32,33,34,35]. Furthermore, according to the research of Chen et al., tumor necrosis factor-α (TNF-α) plays a key role in JEV-induced neuronal death [36]. The inhibitors of TNF (such as lenalidomide and adalimumab) may also have a potential effect on the treatment of JEV infection, which is consistent with the mechanism underlying the treatment of etanercept against JEV infection. Interestingly, these inhibitors were also found in our study. The results suggested that the drugs identified by targeting the top 1% of genes with the GeneRank calculation may be effective in the treatment of JEV infection.

### 2.3. Screening of Genes Associated with JEV Infection by the HotNet2 Algorithm

The HotNet2 (HotNet diffusion-oriented subnetworks) algorithm is based on a heat diffusion kernel algorithm that considers the heats of individual genes as well as the topology of gene-gene interactions. Because the HotNet2 algorithm can reduce the false positive rate, can identify subnetworks with high biological relevance, and can be sensitive to both real and simulated data, it was used to find significant subnetworks associated with various diseases [16]. 

**Figure 4:**
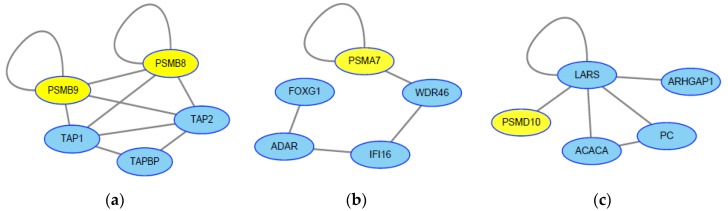
Significant subnetworks associated with encephalitis. (**a**–**c**) represent different subnetworks related to encephalitis. The genes marked by yellow belong to the ubiquitin proteasome system (UPS).

To further screen genes for JEV infection, we applied the HotNet2 algorithm to identify the genes that may contribute to JEV infection. According to the SNP-to-gene mapping method, we mapped the single nucleotide polymorphisms (SNPs) in the phenome-wide association study (PheWAS) data to genes to identify potential genes associated with encephalitis, which exhibits similar symptoms to those of JEV infection [39,40]. To recognize the gene-interaction networks related to encephalitis, we used the *p*-values derived from PheWAS data and the HotNet2 algorithm to calculate the subnetwork. We obtained 16 subnetworks that involved 64 genes associated with encephalitis (Table S3). It should be noted that four genes among the three subnetworks belong to the ubiquitin proteasome system (UPS) (Figure 4), which agrees with the results that encephalitis-related viruses, including JEV, West Nile Virus (WNV), and Venezuelan equine encephalitis virus (VEEV), could utilize the UPS to promote viral entry, replication, and release [41,42,43]. In addition, the proteins (TAP1, TAP2, TAPBP) interacting with PSMB8 and PSMB9 belong to antigen-loading components that were important in the antiviral innate immune response [44]. The protein ADAR in the subnetwork was reported to inhibit hepatitis C virus (HCV) replication through eliminating HCV RNA by adenosine to inosine editing [45]. These results confirmed that the genes identified by the HotNet2 algorithm were important in JEV infection.

### 2.4. Drug Repurposing for JEV Infection by Targeting HotNet2-Derived Genes

By targeting the genes identified by the HotNet2 algorithm, we obtained 20 agents that might have a potential effect on the treatment of JEV infection (Table 2). Interestingly, we found bortezomib among these agents, which was consistent with the agents obtained by the GeneRank calculation. Additionally, the targets of bortezomib belong to the ubiquitin proteasome system, which reinforced our hypothesis that bortezomib may have the ability to treat JEV infection.

In addition to bortezomib, there were other agents that have been reported to have antiviral activity (Table 2). These agents may also be used in the treatment of JEV infection. For example, interferon beta-1A and interferon beta-1B belong to the interferon-I (IFN-I) family, which has antiviral activity and has been reported to treat HCV and Middle East respiratory syndrome coronavirus (MERS-CoV) infections [46,47]. Caffeine has been reported to inhibit HCV replication in vitro at nontoxic concentrations [48]. However, the level of HCV RNA showed no change in patients with long-term caffeine consumption, and the value of IC50 for caffeine to inhibit HCV replication is 0.7263 mM [48,49]. A higher dose of caffeine may be needed to treat HCV infection compared with a regular dose. Doxorubicin, an agent with a broad-spectrum anticancer activity, has been reported to suppress Ebola virus (EBOV) replication in vitro, and it can also inhibit other RNA virus by inducing IFN response [50]. Thus, doxorubicin may also be used in the treatment of JEV infection. Biotin, a B vitamin, can bind to the N protein of porcine epidemic diarrhea virus (PEDV) and inhibit the replication of PEDV in vitro [51]. Since biotin is widely used to bind compounds or proteins to trace them, it is feasible to tag antiviral agents with biotin to improve the antiviral activity.

Furthermore, antibiotics, such as amoxicillin and clavulanate, were also found in our results (Table 2). Considering the fact that JEV infection may also follow bacterial infection and that amoxicillin and clavulanate can be used to relieve inflammation, it may be useful to treat JEV-infected patients with amoxicillin or clavulanate. Interestingly, although there is no evidence for carfilzomib having antiviral activity, the targets and indications of carfilzomib are the same as bortezomib [52]. Therefore, it is possible that carfilzomib has same effect as bortezomib on JEV infection treatment.

### 2.5. Therapeutic Effects of Bortezomib on JEV-Infected Mice

**Figure 5:**
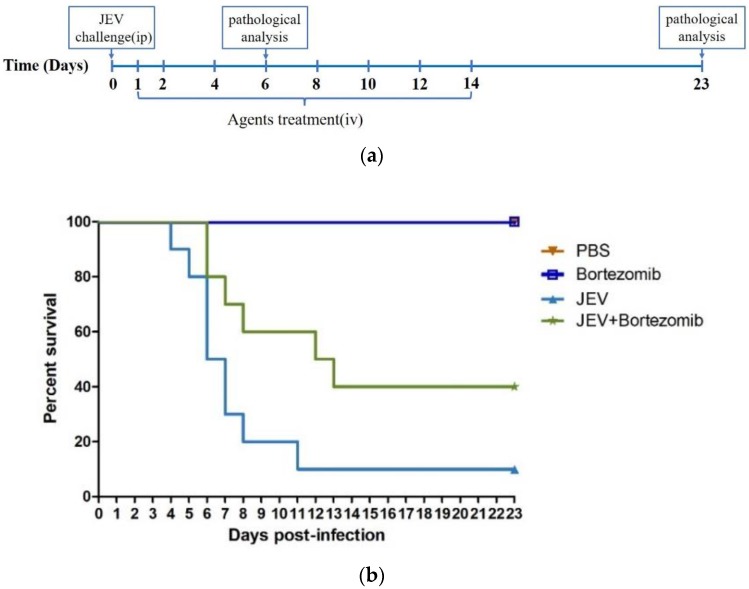

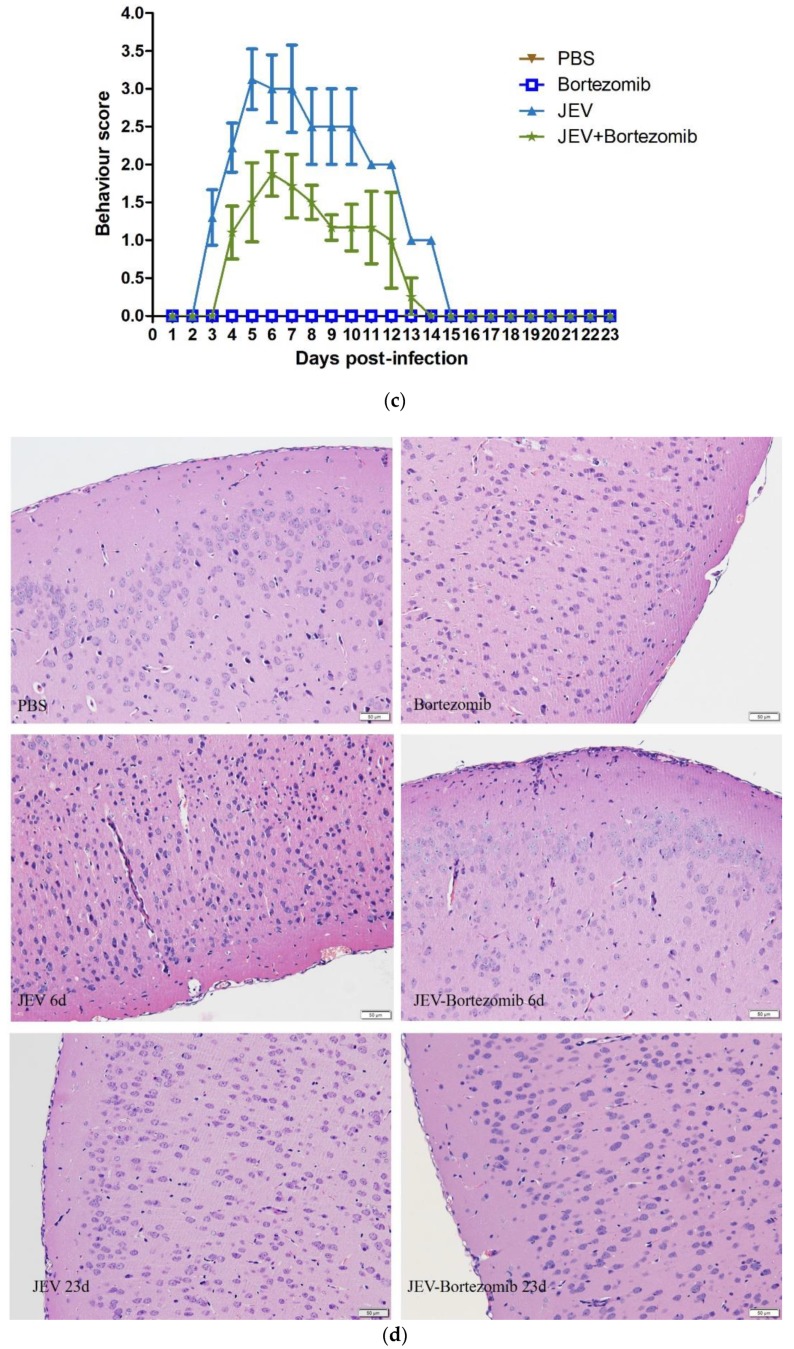
Therapeutic effects of bortezomib on JEV-infected mice. (**a**) Flow chart of animal studies. Mice infected with JEV were treated with PBS or bortezomib (0.5 mg/kg). Brain samples were analyzed on day 6 and day 23 of post-infection. ip: intraperitoneal injection. iv: intravenous injection. (**b**) Survival of mice in each group during the 23 days after JEV infection. Data are shown as Kaplan-Meier survival curves (n = 10 for each group). (**c**) Behavior score of mice in each group during 23 days after JEV infection. 0 = no restriction of movement; no blink frequently; no body stiffening; no hind limb paralysis. 1 = no restriction of movement; blink frequently; no body stiffening; no hind limb paralysis. 2 = restriction of movement; blink frequently; no body stiffening; no hind limb paralysis. 3 = restriction of movement; body stiffening; no hind limb paralysis. 4 = restriction of movement; eyes closed; body stiffening; hind limb paralysis, sometimes tremor. (**d**) Bortezomib reduces the damage in brains caused by JEV infection. Hematoxylin-eosin staining of brain coronal sections was performed to observe the pathological changes.

To further evaluate the above findings that bortezomib has the potential ability to inhibit JEV infection, we established a mouse model of JEV infection. Four-week-old BALB/c mice were randomly divided into four groups: a PBS group; a JEV-infected group; a bortezomib-treated group; and a JEV-infected and bortezomib-treated group. The mice in the infected groups were intraperitoneally injected with 10^6^ PFU of the JEV P3 strain. We administered bortezomib intravenously once every day for the first two days and then administered it every two days (Figure 5a). As anticipated, most mice in the untreated infected group died of JEV infection with a mortality rate of 90%. In contrast, the mortality rate of the bortezomib-treated infected group was 40% (Figure 5b). All of the mice in the bortezomib and PBS groups survived until the end of the experiment, indicating that bortezomib has the ability to protect mice from death caused by JEV infection. 

To verify the effects of bortezomib on clinical symptoms of JEV, we scored the clinical behavior of mice during the experiment [32]. The JEV-infected mice showed different behavior than noninfected mice, including movement limitations, frequent blinking, body stiffening, and hind limb paralysis. The clinical behavior of the bortezomib-treated infected group was alleviated compared with the untreated infected group (Figure 5c), indicating that bortezomib treatment prevented the JEV-infected mice from pain. The mice in the bortezomib and PBS groups did not show any alterations in behavior, suggesting that bortezomib has the potential to alleviate the suffering caused by JEV infection.

Moreover, to further explore the protection of bortezomib against JEV infection in brains, we collected the brain tissues for hematoxylin-eosin (H&E) staining on day 6 and day 23 post infection. As is shown in Figure 5d, the mice in the JEV-infection group suffered from significant meningitis, vacuolar degeneration, and glial nodules, while the symptoms of mice in the bortezomib-treated group were remarkably alleviated. The mice without JEV infection did not show any histological changes, regardless of whether the mice were treated with bortezomib or not. The mice in all groups showed no evidence of meningitis on day 23 post infection. This result indicated that bortezomib could significantly reduce the damage in brains caused by JEV infection. These results further suggested the ability of bortezomib in the treatment of flavivirus infection and confirmed the crucial role of UPS in the lifecycle of flaviviruses. However, as an anticancer agent, bortezomib has many side effects, such as numbness, erythematous plaques or nodules, purpuric eruptions, and folliculitis [53]. Therefore, it is necessary to control the dose in clinical treatment and pay attention to the reaction of patients after taking bortezomib.

## 3. Conclusions

At present, the treatment of JEV infection mainly depends on symptomatic therapy and supportive therapy. Unfortunately, the effect of the existing treatment is far from perfect. Approximately 30–50% of survivors were reported to experience serious sequelae [2]. Although many drugs have been found to have anti-JEV activity, the evaluation of these drugs mainly focused on animal models and cellular levels with few clinical trials reported. Therefore, it is it is necessary to rapidly identify effective therapeutics for JEV infection using the drug repurposing method. Furthermore, since JEV belongs to the same genus as DENV and ZIKV, identifying the agents may provide treatment strategies for those viruses as well.

Identifying the functional genes in JEV infection is essential, not only for finding new antiviral agents but also for understanding the virus replication and pathogenesis. This study utilized the HotNet2 and GeneRank algorithms to identify host genes participating in the progress of JEV infection. We combined the gene expression data with the protein-protein interaction (PPI) database to rank JEV infection-related genes that could be used as the targets to find new antiviral agents. The results showed that host proteins involved in JEV infection include viral infection pathways and immune response-related pathways, which was consistent with the infection mechanism of JEV. Afterwards, we found that bortezomib might be a potential agent for the treatment of JEV infection by targeting these genes. In addition, we identified genetic interaction networks related to encephalitis by the HotNet2 algorithm. Using these genes as the targets to screen drugs, we also found that bortezomib could be used for JEV treatment.

Based on the above results, we confirmed the effect of bortezomib on the treatment of JEV infection in mouse model. Mice treated with bortezomib showed a significant alleviation in histopathological symptoms and clinical symptoms, and a 30% reduction in mortality caused by JEV was observed, compared with the mortality of untreated JEV-infected mice (Figure 5). These results further support the application of host-targeted approaches for new antiviral agents.

Above all, our results provided new insights into the molecular mechanism of JEV infection and offered a novel agent for the treatment of JEV infection.

## 4. Materials and Methods

### 4.1. Data Resources

In this study, the PheWAS data were derived from the work by Denny et al., which included 3144 phenotype-associated single nucleotide polymorphisms (SNPs) [40]. The JEV infection datasets (GEO accession No. GSE57330) came from GEO (www.ncbi.nlm.nih.gov/geo/) [17]. The protein-protein interaction (PPI) network used in the HotNet2 algorithm was obtained from HINT database (http://hint.yulab.org), iRefIndex database (http://irefindex.org), and MultiNet, which included approximately 390,000 interactions [16,54,55,56]. The protein-protein interaction (PPI) network used in the GeneRank algorithm was derived from the STRING database (Version: 10.5, http://string-db.org) [57]. 

Information about the association between chemical agents and its targets was obtained from the Drug-Gene Interaction database (DGIdb, http://dgidb.genome.wustl.edu/), the Therapeutic Target Database (TTD, http://bidd.nus.edu.sg/group/cjttd/), and the DrugBank (http://www.drugbank.ca/).

### 4.2. GeneRank Algorithm

Genes can be ranked by the GeneRank method, based on their expression values and interaction information. The GeneRank algorithm was derived from PageRank [15]. The algorithm is described as follows:

(1) $$r_{j}^{n}=(1-d)ex_{j}+d\sum_{i=1}^{N}\frac{w_{ij}r_{i}{}^{n-1}}{\deg_{i}}$$

where the importance of gene *j* and *i* after *n* or *n* − 1 iterations is represented by $r_{j}^{n}$ and $r_{i}{}^{n-1}$, respectively; the initial importance of gene *j* is represented by $ex_{j}$, $ex_{j}$ is defined as the fold change value in this work; $w_{ij}$ represents the relationship between gene *j* and gene *i* in the PPI network, if gene *i* interacts with gene *j*, then $w_{ij}$ = 1, otherwise $w_{ij}$ = 0; $\deg_{i}$ is the out-degree of gene *i*, which means the number of genes interacting with gene *i*; the total number of genes in the PPI network is represented by *N*; and the parameter *d* (0 ≤ *d* < 1) is a constant representing the proportion of PPI network in calculation. The greater *d* is, the more important PPI network is. In this study, we set the value of *d* to 0.5. 

### 4.3. HotNet2 Algorithm

The HotNet diffusion-oriented subnetworks (HotNet2) algorithm is a topology-based method for finding significant subnetworks associated with disease. Originally, the HotNet2 algorithm was used to analyze somatic mutation data from cancer datasets [16].

The initial input in the HotNet2 algorithm is a heat vector containing the fraction of each gene and a network of protein interactions. At each step, the nodes passed their heat and received heat from adjacent nodes, but also a fraction β (0 ≤ β ≤ 1) of heat was retained. This process runs until equilibrium. Therefore, the heat of each node at equilibrium depends on its initial heat, the local topology of the network around the nodes, and the value β. The process is described as follows:

F = β (I − (1 − β) × W) − 1(2)

where

$$W_{ij}=\{\begin{array}{c} \frac{1}{\deg\left(j\right)}\;\mathrm{if}\;\mathrm{noede}\;i\;\mathrm{interacts}\;\mathrm{with}\;j,\\ 0\;\mathrm{otherwise}. \end{array}$$

where deg(*i*) is the number of neighbors (i.e., the degree) of protein in the interaction network.

In this study, we used the *p*-values of encephalitis derived from PheWAS data as heat scores in the HotNet2 algorithm.

### 4.4. Agents and Virus

Bortezomib (PS-341, powder) was purchased from Selleck Chemicals (Houston, TX, USA). DMSO and PEG300 were purchased from Sigma-Aldrich (St. Louis, MO, USA). JEV P3 strains were kindly provided by Yun-Feng Song, State Key Laboratory of Agricultural Microbiology, Huazhong Agricultural University, China.

### 4.5. Animal Studies

All female BALB/c mice (4-week-old) were purchased from the Hubei Provincial Center for Disease Control and Prevention (Wuhan, China). The mice were randomly divided into four groups: a PBS group (PBS, *n* = 15); a JEV-infected group (JEV, *n* = 15); a bortezomib-treated group (bortezomib, *n* = 15); a JEV-infected and bortezomib-treated group (JEV-bortezomib, *n* = 15). For the JEV-infected group, the mice were intraperitoneally injected with 10^6^ PFU of JEV P3 strain in 100 μL PBS. For the PBS group, mice were intraperitoneally injected with 100 μL PBS. For the bortezomib-treated and vehicle-treated group, mice were intravenously injected with 0.5 mg/kg bortezomib or with PBS with 2% DMSO and 30% PEG 300. 

After JEV infection, the mice were treated with bortezomib once every day for the first two days and were then treated once every two days. On day 6 and day 23 post infection, five mice from each group were euthanized, and the brains were used for subsequent H&E staining. Ten remaining mice were monitored daily to assess behavior and mortality. Behavioral scoring was performed basing on the presence of symptoms [32]. This experiment was approved by the Scientific Ethic Committee of Huazhong Agricultural University (HZAUMO-2017-032).

### 4.6. H&E Staining

For the histology analysis, brain tissues were fixed in 4% paraformaldehyde and were embedded in paraffin. Paraffin sections were stained with hematoxylin-eosin for pathological analysis.

### 4.7. Data Analysis

All statistical analyses were conducted using GraphPad Prism v5.0 (GraphPad Software Inc., San Diego, CA, USA). Cytoscape 3.6.1 was used to visualize the subnetworks. The clusterProfiler, an R package, was used to perform the enrichment analysis of genes.

## Figures and Tables

**Table 1:** Agents reported to have an effect on the treatment of Japanese encephalitis virus (JEV) infection. Among these agents, the effect of minocycline and ribavirin on the treatment for JEV has been tested by randomized clinical trials [37,38]. Etanercept and minocycline inhibited JEV replication both in vitro and in vivo.

Agent	Anti-JEV Potential	Reference
Aspirin	Aspirin suppressed JEV propagation in neuronal and nonneuronal cells	[27]
Chlorpromazine	Chlorpromazine reduced the positive rate of JEV infection by 50% in vitro	[28]
Curcumin	Curcumin inhibited the production of infective JEV particle in vitro	[29]
Etanercept	Etanercept significantly relieved clinical symptoms and reduces mortality in JEV-infected mice	[30]
Genistein	Genistein protected neurons from JEV-induced decrease in the number of visible neurons	[31]
Minocycline	Minocycline protected 70% of mice from JEV-induced death, and inhibited JEV replication in vitro	[32]
Quercetin	Quercetin inhibited JEV replication in vitro	[33]
Ribavirin	Ribavirin inhibited JEV replication in vitro	[34]
Valproic acid	Valproic acid reduced the cytopathic effects caused by JEV	[35]

**Table 2:** Agents targeting the genes identified by the HotNet2 algorithm.

Serial Number	Agents	Indications	Evidence in Antiviral
1	Amoxicillin	bacterial infections	N
2	AT-406	cancer	N
3	Biotin	dietary shortage or imbalance	Y
4	Bortezomib	multiple myeloma, lymphoma	Y
5	Caffeine	fatigue, neurasthenia	Y
6	Carfilzomib	multiple myeloma	N
7	Clavulanate	bacterial infections	N
8	Doxorubicin	various cancer	Y
9	GDC-0152	cancer	N
10	Glatiramer Acetate	multiple sclerosis	N
11	Insulin	diabetes	N
12	Interferon Beta-1A	multiple sclerosis, condyloma acuminatum	Y
13	Interferon Beta-1B	multiple sclerosis	Y
14	*N*-Acetylglucosamine	osteoarthritis	N
15	Niraparib	ovarian cancer, fallopian tube cancer, breast cancer	N
16	Olaparib	ovarian cancer, breast cancer	N
17	Pyruvic Acid	dietary shortage or imbalance	N
18	Rucaparib	ovarian cancer	N
19	Talazoparib	breast cancer	N
20	Veliparib	breast cancer, non-small cell lung cancer	N

## Supplementary Materials

The following are available online. Table S1: The functional genes participating in JEV infection; Table S2: The potential anti-JEV agents discovered by GeneRank algorithm; Table S3: The significant subnetworks associated with encephalitis.

## Abbreviations

DMSO	dimethyl sulfoxide
PBS	phosphate buffer saline
PEG300	polyethylene glycol 300
PFU	plaque forming unit
