# Supplementary material for: Drug Repurposing for Japanese Encephalitis Virus Infection by Systems Biology Methods
Source: Molecules. 2018 Dec 18;23(12):3346. doi: 10.3390/molecules23123346 (PMC6320907; doi:10.3390/molecules23123346)
Supplement: Supplementary file 1 [file molecules-23-03346-s001.zip › supple/table S1.pdf]

Supplementary Table 1: The functional genes participating in JEV infection ranked by GeneRank algorithm

Symbol	Description	GenBank	KEGG pathway
ABCC9	ATP binding cassette subfamily C member 9	NM_005691.3	hsa02010
ABL1			
ACACB	acetyl-CoA carboxylase beta	NM_001093.3	hsa00620, hsa00620, hsa00061, hsa04152, hsa04910, hsa04922, hsa04920, hsa04931
ACLY	ATP citrate lyase	NM_001096.2	hsa00020
ACTA1	actin, alpha 1, skeletal muscle	NM_001100.3	
ACTA2			
ACTB	actin beta	NM_001101.4	hsa04015, hsa04390, hsa04145, hsa04210, hsa04510, hsa04520, hsa04530, hsa04810, hsa04611, hsa04670, hsa04921, hsa04919, hsa04971, hsa04714, hsa05205, hsa05225, hsa05418, hsa05410, hsa05412, hsa05414, hsa05416, hsa05110, hsa05130, hsa05132, hsa05131, hsa05100, hsa05164
ACTBL2			
ACTC1			
ACTG1			
ACTL7A			
ACTL7B			
ACTL9			
ACTN2	actinin alpha 2	NM_001103.3	hsa05412
ACTRT2			
ACVR1C			
ADAMTS17			
ADAR			
ADCY4			
AGTR1			
AKAP9			
AKT1	AKT serine/threonine kinase 1	NM_005163.2	hsa04014, hsa04015, hsa04010, hsa04012, hsa04370, hsa04371, hsa04630, hsa04668, hsa04066, hsa04068, hsa04072, hsa04071, hsa04024, hsa04022, hsa04151, hsa04152, hsa04150, hsa04140, hsa04210, hsa04218, hsa04510, hsa04550, hsa04611, hsa04620, hsa04625, hsa04660, hsa04662, hsa04664, hsa04666, hsa04062, hsa04910, hsa04922, hsa04923, hsa04920, hsa04915, hsa04914, hsa04917, hsa04926, hsa04919, hsa04261, hsa04973, hsa04725, hsa04728, hsa04722, hsa04380, hsa04211, hsa04213, hsa05200, hsa05230, hsa05231, hsa05205, hsa05210, hsa05212, hsa05225, hsa05226, hsa05214, hsa05221, hsa05220, hsa05218, hsa05211, hsa05215, hsa05213, hsa05224, hsa05222, hsa05223, hsa05418, hsa04932, hsa04931, hsa04933, hsa05152, hsa05166, hsa05162, hsa05164, hsa05161, hsa05160, hsa05163, hsa05167, hsa05169, hsa05165, hsa05145, hsa05142, hsa01521, hsa01524, hsa01522
ALB	albumin	NM_000477.6	hsa04918
ALDH1A1			
ANGPT1			
ANKRD20A5			
ANKRD34B			
ANKRD36			
ANXA10			
AP1S2			
APOL2			
APP	amyloid beta precursor protein	NM_000484.3	hsa04726, hsa05010
ARL4A	ADP ribosylation factor like GTPase 4A	NM_005738.4	
ARL5B			
ARRB1	arrestin beta 1	NM_004041.4	hsa04010, hsa04340, hsa04144, hsa04062, hsa04926, hsa04928, hsa04740, hsa05032
ARRB2	arrestin beta 2	NM_004313.3	hsa04010, hsa04340, hsa04144, hsa04062, hsa04926, hsa04928, hsa04740, hsa05032
ASB11			
ASH1L			
ATAD2B			
ATF3	activating transcription factor 3	NM_001674.3	hsa05166
AVPR2			
BAMBI	BMP and activin membrane bound inhibitor	NM_012342.2	hsa04310, hsa04350
BATF2	basic leucine zipper ATF-like transcription factor 2	NM_138456.3	
BCL2	BCL2, apoptosis regulator	NM_000633.2	hsa04141, hsa04340, hsa04630, hsa04064, hsa04066, hsa04071, hsa04151, hsa04140, hsa04210, hsa04215, hsa04217, hsa04115, hsa04510, hsa04621, hsa04915, hsa04928, hsa04261, hsa04725, hsa04722, hsa05200, hsa05206, hsa05210, hsa05226, hsa05215, hsa05222, hsa05014, hsa05418, hsa04933, hsa05152, hsa05161, hsa05169, hsa05145, hsa01521, hsa01524, hsa01522
BCL2A1	BCL2 related protein A1	NM_004049.3	hsa04064, hsa04210, hsa05202, hsa05221
BCL6			
BIRC6			
BMP2			
BST2	bone marrow stromal cell antigen 2	NM_004335.3	
7orf76//LRRC7	Leucine-rich repeat-containing protein 75A	NM_001113567.2	
C1R	complement C1r	NM_001733.6	hsa04145, hsa04610, hsa05322, hsa05133, hsa05150
C1S	complement C1s	NM_201442.3	hsa04610, hsa05322, hsa05133, hsa05150
C3AR1	complement C3a receptor 1	NM_004054.3	hsa04080, hsa04610, hsa05150
CA2			
CACNA1F			
CAD	carbamoyl-phosphate synthetase 2, aspartate transcarba	NM_004341.4	hsa00240, hsa00250
CALM1	calmodulin 1	NM_001363670.1	hsa04014, hsa04015, hsa04371, hsa04020, hsa04070, hsa04024, hsa04022, hsa04114, hsa04218, hsa04625, hsa04910, hsa04922, hsa04912, hsa04915, hsa04921, hsa04916, hsa04924, hsa04925, hsa04261, hsa04270, hsa04970, hsa04971, hsa04728, hsa04720, hsa04722, hsa04744, hsa04740, hsa04750, hsa04713, hsa05200, hsa05214, hsa05010, hsa05031, hsa05034, hsa05418, hsa05133, hsa05152, hsa05163, hsa05167

CALM2	calmodulin 2	NM_001305624.1	hsa04014, hsa04015, hsa04371, hsa04020, hsa04070, hsa04024, hsa04022, hsa04114, hsa04218, hsa04625, hsa04910, hsa04922, hsa04912, hsa04915, hsa04921, hsa04916, hsa04924, hsa04925, hsa04261, hsa04270, hsa04970, hsa04971, hsa04728, hsa04720, hsa04722, hsa04744, hsa04740, hsa04750, hsa04713, hsa05200, hsa05214, hsa05010, hsa05031, hsa05034, hsa05418, hsa05133, hsa05152, hsa05163, hsa05167
CALM3	calmodulin 3	NM_005184.3	hsa04014, hsa04015, hsa04371, hsa04020, hsa04070, hsa04024, hsa04022, hsa04114, hsa04218, hsa04625, hsa04910, hsa04922, hsa04912, hsa04915, hsa04921, hsa04916, hsa04924, hsa04925, hsa04261, hsa04270, hsa04970, hsa04971, hsa04728, hsa04720, hsa04722, hsa04744, hsa04740, hsa04750, hsa04713, hsa05200, hsa05214, hsa05010, hsa05031, hsa05034, hsa05418, hsa05133, hsa05152, hsa05163, hsa05167
CASP1	caspase 1	NM_001257118.2	hsa04217, hsa04621, hsa04623, hsa04625, hsa05014, hsa05132, hsa05133, hsa05134, hsa05164
CASP3	caspase 3	NM_004346.3	hsa04010, hsa04668, hsa04210, hsa04215, hsa04115, hsa04650, hsa04657, hsa04726, hsa05200, hsa05206, hsa05205, hsa05203, hsa05210, hsa05222, hsa05010, hsa05012, hsa05014, hsa05016, hsa05416, hsa04932, hsa04933, hsa05120, hsa05133, hsa05134, hsa05152, hsa05161, hsa05168, hsa05163, hsa05167, hsa05165, hsa05146, hsa05145, hsa01524
CASP5			
CAT			
CBL			
CCDC85A			
CCL20	C-C motif chemokine ligand 20	NM_004591.2	hsa04668, hsa04060, hsa04657, hsa04062, hsa05323
CCL26	C-C motif chemokine ligand 26	NM_006072.4	hsa04060, hsa04062
CCL5	C-C motif chemokine ligand 5	NM_002985.2	hsa04668, hsa04060, hsa4620, hsa04621, hsa04623, hsa04062, hsa05323, hsa05020, hsa05120, hsa05164, hsa05168, hsa05163, hsa05142
CCND1			
CCNT1			
CD274	CD274 molecule	NM_014143.3	hsa04514
CD40LG			
CD44			
CD68			
CDC20			
CDC25C			
CDC40			
CDC42	cell division cycle 42	NM_001791.3	hsa04014, hsa04015, hsa04010, hsa04370, hsa04144, hsa04510, hsa04520, hsa04530, hsa04810, hsa04660, hsa04666, hsa04670, hsa04062, hsa04912, hsa04722, hsa04360, hsa05200, hsa05205, hsa05203, hsa05212, hsa05211, hsa04932, hsa04933, hsa05120, hsa05130, hsa05132, hsa05131, hsa05100, hsa05165
CDH1	cadherin 1	NM_004360.4	hsa04015, hsa04390, hsa04371, hsa04514, hsa04520, hsa05200, hsa05226, hsa05216, hsa05218, hsa05219, hsa05213, hsa05130, hsa05100
CDK1			
CDK2			
CFB	complement factor B	NM_001710.5	hsa04610, hsa05150
CFTR			
CGA			
CH25H	cholesterol 25-hydroxylase	NM_003956.3	hsa00120
CHRNA1			
CMPK2	cytidine/uridine monophosphate kinase 2	NM_207315.3	hsa00240
CNDP1			
CNTF			
COL17A1			
COL1A1			
CREB1			
CREBBP			
CSF2			
CSTA			
CTAGE3P			
CTNNB1	catenin beta 1	NM_001904.3	hsa04015, hsa04310, hsa04390, hsa04510, hsa04520, hsa04550, hsa04670, hsa04919, hsa04916, hsa05200, hsa05205, hsa05210, hsa05225, hsa05226, hsa05216, hsa05217, hsa05215, hsa05213, hsa05224, hsa05418, hsa05412, hsa04934, hsa05130, hsa05100, hsa05166, hsa05163, hsa05167, hsa05165
CTSS	cathepsin S	NM_004079.4	hsa04145, hsa04142, hsa04210, hsa04612, hsa05152
CXCL10	C-X-C motif chemokine ligand 10	NM_001565.3	hsa04668, hsa04060, has04620, hsa04622, hsa04623,hsa04657, hsa04062, hsa05164
CXCL11	C-X-C motif chemokine ligand 11	NM_005409.4	hsa04060, hsa04620, hsa04062
CXCL3			
CXCR4			
CYCS			
CYLC1			
DCAF17			
DDIT4			
DDO	D-aspartate oxidase	NM_004032.2	hsa00250, hsa04146
DDX58	DExD/H-box helicase 58	NM_014314.3	hsa04064, hsa04622, hsa04623, hsa05162, hsa05164, hsa05161, hsa05160, hsa05168, hsa05169, hsa04622
DDX60	DExD/H-box helicase 60	NM_017631.5	
DDX60L	DExD/H-box 60 like	NM_001012967.2	
DEFB110			
DHH			
DHX58	DExH-box helicase 58	NM_024119.2	hsa04622
DHX8			
DICER1			
DNAH8			
DNAJB14			
DNAJC10			
DPYD			
DPYS			

DTX3L	deltex E3 ubiquitin ligase 3L	NM_138287.3	hsa04330
DUOX2			
EDN1			
EGF	epidermal growth factor	NM_001963.5	hsa04014, hsa04015, hsa04010, hsa04012, hsa04630, hsa04066, hsa04068, hsa04072, hsa04151, hsa04510, hsa04540, hsa04810, hsa05200, hsa05231, hsa05210, hsa05212, hsa05226, hsa05214, hsa05218, hsa05219, hsa05215, hsa05213, hsa05224, hsa05223, hsa05160, hsa05165, hsa01521
EGFR	epidermal growth factor receptor	NM_005228.4	hsa04014, hsa04015, hsa04010, hsa04012, hsa04630, hsa04066, hsa04068, hsa04020, hsa04072, hsa04151, hsa04144, hsa04510, hsa04520, hsa04540, hsa04810, hsa04912, hsa04915, hsa04921, hsa04926, hsa04928, hsa05200, hsa05230, hsa05231, hsa05206, hsa05205, hsa05210, hsa05212, hsa05225, hsa05226, hsa05214, hsa05218, hsa05219, hsa05215, hsa05213, hsa05224, hsa05223, hsa04934, hsa05120, hsa05160, hsa05163, hsa05165, hsa01521, hsa01522
EGR1			
EHHADH			
EHMT1	euchromatic histone lysine methyltransferase 1	NM_024757.4	hsa00310, hsa04211
EHMT2	euchromatic histone lysine methyltransferase 2	NM_001289413.1	hsa00310, hsa04211
EID3			
EIF2AK2	eukaryotic translation initiation factor 2 alpha kinase 2	NM_002759.3	hsa04141, hsa04217, hsa05203, hsa05162, hsa05164, hsa05160, hsa05168, hsa05167, hsa05169, hsa05165
EML6			
ENO2			
EP300	E1A binding protein p300	NM_001429.3	hsa04310, hsa04330, hsa04350, hsa04630, hsa04066, hsa04068, hsa04024, hsa04110, hsa04520, hsa04922, hsa04919, hsa04916, hsa04720, hsa05200, hsa05206, hsa05203, hsa05211, hsa05215, hsa05016, hsa05152, hsa05166, hsa05164, hsa05161, hsa05168, hsa05167, hsa05169, hsa05165
EPRS			
EPSTI1	epithelial stromal interaction 1	NM_001002264.3	
ESM1	endothelial cell specific molecule 1	NM_007036.4	
ESR1	estrogen receptor 1	NM_000125.3	hsa04915, hsa04917, hsa04919, hsa04961, hsa05200, hsa05205, hsa05224, hsa01522
ETV7			
EZH2			
F2R			
F3	coagulation factor III, tissue factor	NM_001993.4	hsa04610, hsa04933
FAM183B			
FAM43A	family with sequence similarity 43 member A	NM_153690.4	
FAM46A	terminal nucleotidyltransferase 5A	NM_017633.2	
FAM83E			
FAS	Fas cell surface death receptor	NM_000043.5	hsa04010, hsa04668, hsa04060, hsa04210, hsa04217, hsa04115, hsa04650, hsa05200, hsa05205, hsa05320, hsa05330, hsa05332, hsa05010, hsa04940, hsa04932, hsa05162, hsa05164, hsa05161, hsa05168, hsa05163, hsa05167, hsa05165, hsa05142, hsa05143, hsa01524
FCGR3B			
FGF14			
FGF2	fibroblast growth factor 2	NM_002006.5	hsa04014, hsa04015, hsa04010, hsa04151, hsa04550, hsa04810, hsa05200, hsa05205, hsa05226, hsa05218, hsa05224, hsa05167, hsa01521
FLJ42393			
FLNA			
FOS			
FST	follistatin (FST)	NM_006350.3	hsa04350
FURIN			
FYN			
GAPDH	glyceraldehyde-3-phosphate dehydrogenase	NM_002046.6	hsa00010, hsa04066, hsa05010
GART	phosphoribosylaminoimidazole synthetase (GART)	NM_000819.4	hsa00230, hsa00670, hsa01523
GAS2L3			
GBP1	guanylate binding protein 1	NM_002053.2	hsa04621
GBP2	guanylate binding protein 2	NM_004120.4	hsa04621
GBP3	guanylate binding protein 3	NM_018284.2	hsa04621
GBP5	guanylate binding protein 5	NM_001134486.2	hsa04621
GLDC			
GLS			
GMPR	guanosine monophosphate reductase	NM_006877.3	hsa00230
GMPS	guanine monophosphate synthase	NM_003875.2	hsa00230, hsa00983
GNAI2			
GNAL	G protein subunit alpha L	NM_182978.3	hsa04020, hsa04728, hsa04740, hsa05012, hsa05146, hsa05142
GNAT3			
GNB2			
GNB4	G protein subunit beta 4	NM_021629.3	hsa04014, hsa04371, hsa04151, hsa04062, hsa04926, hsa04724, hsa04727, hsa04725, hsa04728, hsa04726, hsa04723, hsa04713, hsa05200, hsa05032, hsa05034, hsa05163, hsa05167
GNG13	G protein subunit gamma 13	NM_016541.2	hsa04014, hsa04371, hsa04151, hsa04062, hsa04926, hsa04724, hsa04727, hsa04725, hsa04728, hsa04726, hsa04723, hsa04740, hsa04742, hsa04713, hsa05200, hsa05032, hsa05034, hsa05163, hsa05167
GNG4			
GNG7	G protein subunit gamma 7	NM_052847.2	hsa04014, hsa04371, hsa04151, hsa04062, hsa04926, hsa04724, hsa04727, hsa04725, hsa04728, hsa04726, hsa04723, hsa04740, hsa04713, hsa05200, hsa05032, hsa05034, hsa05163, hsa05167
GNGT1	G protein subunit gamma transducin 1	NM_021955.4	hsa04014, hsa04371, hsa04151, hsa04062, hsa04926, hsa04724, hsa04727, hsa04725, hsa04728, hsa04726, hsa04723, hsa04744, hsa04713, hsa05200, hsa05032, hsa05034, hsa05163, hsa05167
GPR37			
GRB2			
GRP			
GSK3B			
H2AFB2			
H2AFV			
HACE1			

HAO1			
HAS2	hyaluronan synthase 2	NM_005328.2	
HCK			
HDAC1			
HDAC2			
HDX			
HECW2			
HERC5	HECT and RLD domain containing E3 ubiquitin protei	NM_016323.3	
HERC6	HECT and RLD domain containing E3 ubiquitin protei	NM_017912.3	
HIST1H2AA			
HIST1H2BA			
HIST1H2BL			
HIST2H2AC			
HIST2H2BE			
HLA-A	major histocompatibility complex, class I, A	NM_002116.7	hsa04514, hsa04144, hsa04145, hsa04218, hsa04650, hsa04612, hsa05203, hsa05320, hsa05330, hsa05332, hsa05416, hsa04940, hsa05166, hsa05168, hsa05163, hsa05167, hsa05169, hsa05165
HLA-B	major histocompatibility complex, class I, B (HLA-B)	NM_005514.7	hsa04514, hsa04144, hsa04145, hsa04218, hsa04650, hsa04612, hsa05203, hsa05320, hsa05330, hsa05332, hsa05416, hsa04940, hsa05166, hsa05168, hsa05163, hsa05167, hsa05169, hsa05165
ILA-B /// HLA-(	major histocompatibility complex, class I, B	NM_005514.7	hsa04514, hsa04144, hsa04145, hsa04218, hsa04650, hsa04612, hsa05203, hsa05320, hsa05330, hsa05332, hsa05416, hsa04940, hsa05166, hsa05168, hsa05163, hsa05167, hsa05169, hsa05165
HLA-C	major histocompatibility complex, class I, C	NM_002117.5	hsa04514, hsa04144, hsa04145, hsa04218, hsa04650, hsa04612, hsa05203, hsa05320, hsa05330, hsa05332, hsa05416, hsa04940, hsa05166, hsa05168, hsa05163, hsa05167, hsa05169, hsa05165
HLA-E			
HLA-F	major histocompatibility complex, class I, F	NM_001098479.1	hsa04514, hsa04144, hsa04145, hsa04218, hsa04650, hsa04612, hsa05203, hsa05320, hsa05330, hsa05332, hsa05416, hsa04940, hsa05166, hsa05168, hsa05163, hsa05167, hsa05169, hsa05165
HMSD			
HOXA10			
HRAS	HRas proto-oncogene, GTPase	NM_005343.3	hsa04014, hsa04015, hsa04010, hsa04012, hsa04370, hsa04371, hsa04630, hsa04068, hsa04072, hsa04071, hsa04151, hsa04150, hsa04144, hsa04140, hsa04137, hsa04210, hsa04218, hsa04510, hsa04540, hsa04550, hsa04810, hsa04625, hsa04650, hsa04660, hsa04662, hsa04664, hsa04062, hsa04910, hsa04912, hsa04915, hsa04917, hsa04921, hsa04926, hsa04919, hsa04916, hsa04725, hsa04726, hsa04720, hsa04730, hsa04722, hsa04360, hsa04211, hsa04213, hsa04714, hsa05200, hsa05230, hsa05231, hsa05206, hsa05205, hsa05203, hsa05210, hsa05225, hsa05226, hsa05214, hsa05216, hsa05221, hsa05220, hsa05218, hsa05211, hsa05219, hsa05215, hsa05213, hsa05224, hsa05223, hsa05034, hsa04933, hsa05166, hsa05161, hsa05160, hsa05163, hsa05167, hsa05165, hsa01521, hsa01522
HSP90AA1	heat shock protein 90 alpha family class A member 1	NM_001017963.2	hsa04141, hsa04151, hsa04217, hsa04621, hsa04612, hsa04659, hsa04657, hsa04915, hsa04914, hsa05200, hsa05215, hsa05418
HSP90AB1			
HSPA1A			
HSPA4			
HSPA4L			
HSPA5			
HSPA6	heat shock protein family A (Hsp70) member 6	NM_002155.4	hsa03040, hsa04141, hsa04010, hsa04144, hsa04612, hsa04915, hsa04213, hsa05134, hsa05162, hsa05164, hsa05169, hsa05145
ISPA6 /// HSPA	heat shock protein family A (Hsp70) member 6	NM_002155.4	hsa03040, hsa04141, hsa04010, hsa04144, hsa04612, hsa04915, hsa04213, hsa05134, hsa05162, hsa05164, hsa05169, hsa05145
HSPA8			
HSPB3			
HTR2B			
ICAM1			
IDO1	indoleamine 2,3-dioxygenase 1	NM_002164.5	hsa00380, hsa05143
IFI16	interferon gamma inducible protein 16	NM_001206567.1	hsa047621
IFI27	interferon alpha inducible protein 27	NM_001130080.2	
IFI35	interferon induced protein 35	NM_005533.4	
IFI44	interferon induced protein 44	NM_006417.4	
IFI44L	interferon induced protein 44 like	NM_006820.3	
IFI6	interferon alpha inducible protein 6	NM_002038.3	
IFIH1	interferon induced with helicase C domain 1	NM_022168.4	
IFIT1	interferon induced protein with tetratricopeptide repeat	NM_001548.4	hsa05160, hsa05168
IFIT2	interferon induced protein with tetratricopeptide repeat	NM_001547.4	
IFIT3	interferon induced protein with tetratricopeptide repeat	NM_001549.5	
IFIT5	interferon induced protein with tetratricopeptide repeat	NM_012420.2	
IFITM1	interferon induced transmembrane protein 1	NM_003641.3	hsa04662
IFNA10			
IFNA16	interferon alpha 16	NM_002173.3	hsa04630, hsa04151, hsa04060, hsa04217, hsa04620, hsa04621, hsa04622, hsa04623, hsa04650, hsa05200, hsa05320, hsa05152, hsa05162, hsa05164, hsa05161, hsa05160, hsa05168, hsa05163, hsa05167, hsa05165
IFNA17	interferon alpha 17	NM_021268.2	hsa04630, hsa04151, hsa04060, hsa04217, hsa04620, hsa04621, hsa04622, hsa04623, hsa04650, hsa05200, hsa05320, hsa05152, hsa05162, hsa05164, hsa05161, hsa05160, hsa05168, hsa05163, hsa05167, hsa05165
IFNB1	interferon beta 1	NM_002176.3	hsa04630, hsa04668, hsa04151, hsa04060, hsa04217, hsa04620, hsa04621,hsa04622, hsa04623, hsa04650, hsa04380, hsa05152, hsa05162, hsa05164, hsa05161, hsa05160, hsa05168, hsa05163, hsa05167, hsa05165, hsa05142
IFNG			
IGF1			
IGLL3P			
IL10			
IL15	interleukin 15	NM_172175.2	hsa04630, hsa04668, hsa04060, hsa04672, hsa05200, hsa05323, hsa05166, hsa05168
IL18			

IL1B	interleukin 1-beta	NM_000576.2	hsa04010, hsa04064, hsa04668, hsa04060, hsa04217, hsa04640, hsa04620, hsa04621, hsa04623, hsa04625, hsa04659, hsa04657, hsa04750, hsa04380, hsa05323, hsa05321, hsa05332, hsa05010, hsa05020, hsa05418, hsa04940, hsa04932, hsa04933, hsa05132, hsa05133, hsa05134, hsa05152, hsa05162, hsa05164, hsa05168, hsa05163, hsa05146, hsa05144, hsa05140, hsa05142, hsa05143, hsa01523
IL2			
IL28A			
IL29	interleukin 29/interferon lambda 1	NM_172140.1	
IL4			
IL4I1			
IL6	interleukin 6	NM_000600.4	hsa04630, hsa04668, hsa04066, hsa04068, hsa04151, hsa04060, hsa04218, hsa04640, hsa04620, hsa04621, hsa04623, hsa04625, hsa04659, hsa04657, hsa04672, hsa05200, hsa05202, hsa05323, hsa05321, hsa05332, hsa05020, hsa05410, hsa04932, hsa04931, hsa04933, hsa05132, hsa05133, hsa05134, hsa05152, hsa05166, hsa05162, hsa05164, hsa05161, hsa05168, hsa05163, hsa05167, hsa05146, hsa05144, hsa05142, hsa05143, hsa01521, hsa01523
IL7R	interleukin 7 receptor	NM_002185.4	hsa04630, hsa04068, hsa04151, hsa04060, hsa04640, hsa05200, hsa05340
IMPDH2	inosine monophosphate dehydrogenase 2	NM_000884.2	hsa00230, hsa00983
INHBE			
IRF1	interferon regulatory factor 1	NM_002198.2	hsa04668, hsa04625, hsa04917, hsa05133, hsa05160, hsa05165
IRF2	interferon regulatory factor 2	NM_002199.3	
IRF7	interferon regulatory factor 7	NM_001572.4	hsa04620, hsa04621, hsa04622, hsa04623, hsa05203, hsa05162, hsa05164, hsa05161, hsa05160, hsa05168, hsa05167
IRF9	interferon regulatory factor 9	NM_006084.4	hsa04630, hsa04217, hsa04621, hsa04625, hsa04380, hsa05203, hsa05162, hsa05164, hsa05160, hsa05168, hsa05167, hsa05165
IRS2			
ISG15	ISG15 ubiquitin-like modifier	NM_005101.4	hsa04622, hsa05165
ISG20	interferon stimulated exonuclease gene 20	NM_002201.5	
ISPD			
ITGA2			
ITGB1			
ITGB2			
JAK1			
JAK2	Janus kinase 2	NM_004972.3	hsa04630, hsa04151, hsa04217, hsa04550, hsa04658, hsa04659, hsa04062, hsa04920, hsa04917, hsa04725, hsa05200, hsa04933, hsa05152, hsa05162, hsa05164, hsa05168, hsa05167, hsa05145, hsa05140, hsa01521
JAKMIP2			
JUN	Jun proto-oncogene, AP-1 transcription factor subunit	NM_002228.3	hsa04010, hsa04012, hsa04310, hsa04668, hsa04024, hsa04137, hsa04210, hsa04510, hsa04530, hsa04620, hsa04621, hsa04625, hsa04660, hsa04658, hsa04659, hsa04657, hsa04662, hsa04912, hsa04915, hsa04921, hsa04926, hsa04722, hsa04380, hsa05200, hsa05231, hsa05203, hsa05210, hsa05211, hsa05224, hsa05323, hsa05321, hsa05030, hsa05031, hsa05418, hsa04932, hsa04933, hsa05120, hsa05132, hsa05133, hsa05166, hsa05164, hsa05161, hsa05168, hsa05167, hsa05169, hsa05140, hsa05142, hsa01522
KCNQ1OT1			
KCNV1	potassium voltage-gated channel modifier subfamily V	NM_014379.3	
KIAA0319			
KIAA0754			
KIT	KIT proto-oncogene receptor tyrosine kinase	NM_000222.2	hsa04014, hsa04015, hsa04010, hsa04072, hsa04151, hsa04640, hsa04916, hsa05200, hsa05230, hsa05221, hsa05224
KLF4	Kruppel like factor 4	NM_001314052.1	hsa04550
KLHDC7A			
KRAS			
KRTAP17-1			
KRTAP19-2			
KRTAP21-3			
KRTAP5-10			
KRTAP5-3			
KYNU	kynureninase	NM_003937.2	hsa00380
LAP3	leucine aminopeptidase 3	NM_015907.2	hsa00330, hsa00480
LBX1			
LCN2			
LEUTX			
LMO2			
LRGUK	leucine rich repeats and guanylate kinase domain conta	NM_001365700.1	
LRRK2	leucine rich repeat kinase 2	NM_198578.3	hsa00010
LRRN3	leucine rich repeat neuronal 3	NM_001099660.1	
LYN			
LYPD1	LY6/PLAUR domain containing 1	NM_144586.6	
MAPK1	mitogen-activated protein kinase 1	NM_002745.4	hsa04014, hsa04015, hsa04010, hsa04012, hsa04350, hsa04370, hsa04371, hsa04668, hsa04066, hsa04068, hsa04072, hsa04071, hsa04024, hsa04022, hsa04151, hsa04150, hsa04140, hsa04114, hsa04210, hsa04218, hsa04510, hsa04520, hsa04540, hsa04550, hsa04810, hsa04611, hsa04620, hsa04621, hsa04625, hsa04650, hsa04660, hsa04658, hsa04659, hsa04657, hsa04662, hsa04664, hsa04666, hsa04062, hsa04910, hsa04912, hsa04915, hsa04914, hsa04917, hsa04921, hsa04926, hsa04919, hsa04928, hsa04916, hsa04261, hsa04270, hsa04960, hsa04724, hsa04725, hsa04726, hsa04720, hsa04730, hsa04723, hsa04722, hsa04360, hsa04380, hsa04713, hsa05200, hsa05230, hsa05231, hsa05206, hsa05205, hsa05203, hsa05210, hsa05212, hsa05225, hsa05226, hsa05214, hsa05216, hsa05221, hsa05220, hsa05218, hsa05211, hsa05219, hsa05215, hsa05213, hsa05224, hsa05223, hsa05010, hsa05020, hsa05034, hsa04930, hsa04933, hsa04934, hsa05132, hsa05131, hsa05133, hsa05152, hsa05164, hsa05161, hsa05160, hsa05163, hsa05167, hsa05165, hsa05145, hsa05140, hsa05142, hsa01521, hsa01524, hsa01522
MAPK11			
MAPK12			

MAPK14	mitogen-activated protein kinase 14	NM_001315.2	hsa04015, hsa04010, hsa04370, hsa04668, hsa04068, hsa04071, hsa04218, hsa04550, hsa04611, hsa04620, hsa04621, hsa04622, hsa04625, hsa04660, hsa04658, hsa04659, hsa04657, hsa04664, hsa04670, hsa04912, hsa04914, hsa04917, hsa04926, hsa04261, hsa04728, hsa04723, hsa04722, hsa04750, hsa04380, hsa04714, hsa05205, hsa05014, hsa05418, hsa04933, hsa05120, hsa05132, hsa05131, hsa05133, hsa05152, hsa05164, hsa05160, hsa05163, hsa05167, hsa05169, hsa05145, hsa05140, hsa05142, hsa01522
MAPK3	mitogen-activated protein kinase 3	NM_002746.2	hsa04014, hsa04015, hsa04010, hsa04012, hsa04350, hsa04370, hsa04371, hsa04668, hsa04066, hsa04068, hsa04072, hsa04071, hsa04024, hsa04022, hsa04151, hsa04150, hsa04140, hsa04114, hsa04210, hsa04218, hsa04510, hsa04520, hsa04540, hsa04550, hsa04810, hsa04611, hsa04620, hsa04621, hsa04625, hsa04650, hsa04660, hsa04658, hsa04659, hsa04657, hsa04662, hsa04664, hsa04666, hsa04062, hsa04910, hsa04912, hsa04915, hsa04914, hsa04917, hsa04921, hsa04926, hsa04919, hsa04928, hsa04916, hsa04261, hsa04270, hsa04960, hsa04724, hsa04725, hsa04726, hsa04720, hsa04730, hsa04723, hsa04722, hsa04360, hsa04380, hsa04713, hsa05200, hsa05230, hsa05231, hsa05205, hsa05203, hsa05210, hsa05212, hsa05225, hsa05226, hsa05214, hsa05216, hsa05221, hsa05220, hsa05218, hsa05211, hsa05219, hsa05215, hsa05213, hsa05224, hsa05223, hsa05010, hsa05020, hsa05034, hsa04930, hsa04933, hsa04934, hsa05132, hsa05131, hsa05133, hsa05152, hsa05164, hsa05161, hsa05160, hsa05163, hsa05167, hsa05165, hsa05145, hsa05140, hsa05142, hsa01521, hsa01524, hsa01522
MAPK8			
MARCO			
MBOAT4	membrane bound O-acyltransferase domain containing	NM_001100916.1	
MGP			
MIB1			
MIB2			
MMP1	matrix metallopeptidase 1	NM_002421.3	hsa04657, hsa03320, hsa04926, hsa05200, hsa05219, hsa05323
MMP2			
MS4A3			
MTHFR			
MTMR8			
MTOR			
MUSK			
MX1	MX dynamin like GTPase 1	NM_001144925.2	hsa05162, hsa05164, hsa05165
MX2	MX dynamin like GTPase 2	NM_002463.1	
MYC	MYC proto-oncogene, bHLH transcription factor	NM_002467.5	hsa04010, hsa04012, hsa04310, hsa04350, hsa04390, hsa04630, hsa04151, hsa04110, hsa04218, hsa04550, hsa04919, hsa05200, hsa05230, hsa05202, hsa05206, hsa05205, hsa05210, hsa05225, hsa05226, hsa05216, hsa05221, hsa05220, hsa05219, hsa05213, hsa05224, hsa05222, hsa05166, hsa05161, hsa05163, hsa05167, hsa05169
MYD88			
MYH14			
MYL2			
NAA16			
NANOG			
NCAM1			
NCF2	neutrophil cytosolic factor 2	NM_000433.3	hsa04145, hsa04670, hsa04380, hsa05418, hsa05140
NCOA7			
NCRNA00189			
NEDD9	neural precursor cell expressed, developmentally down	NM_006403.3	
NEURL1B			
NFKB1			
NFKBIA	NFKB inhibitor alpha	NM_020529.2	hsa04064, hsa04668, hsa04024, hsa04210, hsa04620, hsa04621, hsa04622, hsa04623, hsa04625, hsa04660, hsa04658, hsa04659, hsa04657, hsa04662, hsa04062, hsa04920, hsa04926, hsa04722, hsa04380, hsa05200, hsa05203, hsa05220, hsa05215, hsa05222, hsa04931, hsa05120, hsa05131, hsa05134, hsa05166, hsa05162, hsa05164, hsa05161, hsa05160, hsa05168, hsa05163, hsa05167, hsa05169, hsa05145, hsa05140, hsa05142
NIPSNAP3B			
NLRC5	NLR family CARD domain containing 5	NM_032206.4	
NMI	N-myc and STAT interactor	NM_004688.2	
NOL4	nucleolar protein 4	NM_003787.4	
NOTCH1	notch 1	NM_017617.5	hsa04330, hsa04658, hsa04919, hsa05200, hsa05206, hsa05224, hsa05020, hsa05165, hsa01522
NPPC			
NPS			
NR2F1	nuclear receptor subfamily 2 group F member 1	NM_005654.5	
NR4A2	nuclear receptor subfamily 4 group A member 2	NM_006186.3	hsa04928, hsa04925
NTS			
OAS1	2'-5'-oligoadenylate synthetase 1	NM_016816.3	hsa04621, has05162, hsa05164, hsa05160, hsa05168
OAS2	2'-5'-oligoadenylate synthetase 2	NM_016817.2	hsa04621, has05162, hsa05164, hsa05160, hsa05168
OAS3	2'-5'-oligoadenylate synthetase 3	NM_006187.3	hsa04621, has05162, hsa05164, hsa05160, hsa05168
OASL	2'-5'-oligoadenylate synthetase like	NM_003733.3	hsa05165
OR4K2			
OR5AK2			
P2RY12			
PARP12	poly(ADP-ribose) polymerase family member 12	NM_022750.3	
PARP14	poly(ADP-ribose) polymerase family member 14	NM_017554.2	
PARP9	poly(ADP-ribose) polymerase family member 9	NM_031458.2	
PCDH17	protocadherin 17	NM_001040429.2	
PCGF5			
PCNA			
PDE6C			
PDE9A			
PHF11	PHD finger protein 11	NM_001040443.2	
PHLPP1	PH domain and leucine rich repeat protein phosphatas	NM_194449.3	hsa04151

PIK3CA	phosphatidylinositol-4,5-bisphosphate 3-kinase catalyt	NM_006218.3	hsa00562, hsa04014, hsa04015, hsa04012, hsa04370, hsa04630, hsa04668, hsa04066, hsa04068 , hsa04070, hsa04072, hsa04071, hsa04024, hsa04151, hsa04152, hsa04150, hsa04140, hsa04210, hsa04218, hsa04510, hsa04550, hsa04810, hsa04611, hsa04620, hsa04625, hsa04650, hsa04660, hsa04662, hsa04664, hsa04666, hsa04670, hsa04062, hsa04910, hsa04923, hsa04915, hsa04914, hsa04917, hsa04926, hsa04919, hsa04973, hsa04960, hsa04725, hsa04722, hsa04750, hsa04360, hsa04380, hsa04211, hsa04213, hsa05200, hsa05230, hsa05231, hsa05206,hsa05205, hsa05203, hsa05210, hsa05212, hsa05225, hsa05226, hsa05214, hsa05221, hsa05220, hsa05218, hsa05211, hsa05215, hsa05213, hsa05224, hsa05222, hsa05223, hsa05418, hsa04930, hsa04932, hsa04931, hsa04933, hsa05100, hsa05166, hsa05162, hsa05164, hsa05161, hsa05160, hsa05163, hsa05167, hsa05169, hsa05165, hsa05146, hsa05142, hsa01521, hsa01524, hsa01522
PIK3CB	phosphatidylinositol-4,5-bisphosphate 3-kinase catalyt	NM_006219.2	hsa00562, hsa04014, hsa04015, hsa04012, hsa04370, hsa04630, hsa04668, hsa04066, hsa04068 , hsa04070, hsa04072, hsa04071, hsa04024, hsa04151, hsa04152, hsa04150, hsa04140, hsa04210, hsa04218, hsa04510, hsa04550, hsa04810, hsa04611, hsa04620, hsa04625, hsa04650, hsa04660, hsa04662, hsa04664, hsa04666, hsa04670, hsa04062, hsa04910, hsa04923, hsa04915, hsa04914, hsa04917, hsa04926, hsa04919, hsa04973, hsa04960, hsa04725, hsa04722, hsa04750, hsa04360, hsa04380, hsa04211, hsa04213, hsa05200, hsa05230, hsa05231, hsa05205, hsa05203, hsa05210, hsa05212, hsa05225, hsa05226, hsa05214, hsa05221, hsa05220, hsa05218, hsa05211, hsa05215, hsa05213, hsa05224, hsa05222, hsa05223, hsa05418, hsa04930, hsa04932, hsa04931, hsa04933, hsa05100, hsa05166, hsa05162, hsa05164, hsa05161, hsa05160, hsa05163, hsa05167, hsa05169, hsa05165, hsa05146, hsa05142, hsa01521, hsa01524, hsa01522
PIK3CD			
PIK3CG	phosphatidylinositol-4,5-bisphosphate 3-kinase catalyt	NM_002649.3	hsa00562, hsa04371, hsa04072, hsa04022, hsa04151, hsa04611, hsa04921, hsa04261, hsa04725, hsa05167, hsa05145
PIKFYVE	phosphoinositide kinase, FYVE-type zinc finger contai	NM_015040.3	hsa00562, hsa04070, hsa04145, hsa04810
PKN1			
PLA2G4A	phospholipase A2 group IVA	NM_024420.2	hsa00564, hsa00565, hsa00590, hsa00591, hsa00592, hsa04014, hsa04010, hsa04370, hsa04072, hsa04217, hsa04611, hsa04664, hsa04666, hsa04912, hsa04913, hsa04921, hsa04270, hsa04724, hsa04726, hsa04730, hsa04750, hsa05231
PLCL2			
PLEK			
PLEKHA4	pleckstrin homology domain containing A4	NM_020904.2	
PLG			
PLK1			
PLSCR1	phospholipid scramblase 1	NM_021105.2	
PLSCR2			
PMAIP1	phorbol-12-myristate-13-acetate-induced protein 1	NM_021127.2	hsa04210, hsa04215, hsa04115, hsa05200, hsa05203, hsa05210, hsa01524
PMS2CL			
PNMAL1			
PNPLA6			
PNPT1			
POM121L8P			
POMC			
PPARG			
PPBP			
PPM1K	protein phosphatase Mg2+/Mn2+ dependent 1K	NM_152542.4	
PPP1R15A	protein phosphatase 1 regulatory subunit 15A	NM_014330.3	hsa04141
PPP2CA			
PPP2R1A			
PRAMEF12			
PRDM10	PR/SET domain 10	NM_020228.2	
PRKACA	protein kinase cAMP-activated catalytic subunit alpha	NM_002730.3	hsa04014, hsa04010, hsa04310, hsa04340, hsa04371, hsa04020, hsa04024, hsa04140, hsa04114, hsa04530, hsa04540, hsa04611, hsa04062, hsa04911, hsa04910, hsa04922, hsa04923, hsa04912, hsa04913, hsa04915, hsa04914, hsa04921, hsa04926, hsa04918, hsa04919, hsa04928, hsa04916, hsa04924, hsa04925, hsa04927, hsa04261, hsa04270, hsa04970, hsa04971, hsa04976, hsa04962, hsa04961, hsa04724, hsa04727, hsa04725, hsa04728, hsa04726, hsa04720, hsa04723, hsa04740, hsa04742, hsa04750, hsa04211, hsa04213, hsa04713, hsa04714, hsa05200, hsa05205, hsa05203, hsa05012, hsa05020, hsa05030, hsa05031, hsa05032, hsa05034, hsa05414, hsa04934, hsa05110, hsa05166, hsa05163, hsa05169, hsa05165, hsa05146, hsa01522
PRKACB	protein kinase cAMP-activated catalytic subunit beta	NM_182948.3	hsa04014, hsa04010, hsa04310, hsa04340, hsa04371, hsa04020, hsa04024, hsa04140, hsa04114, hsa04530, hsa04540, hsa04611, hsa04062, hsa04911, hsa04910, hsa04922, hsa04923, hsa04912, hsa04913, hsa04915, hsa04914, hsa04921, hsa04926, hsa04918, hsa04919, hsa04928, hsa04916, hsa04924, hsa04925, hsa04927, hsa04261, hsa04270, hsa04970, hsa04971, hsa04976, hsa04962, hsa04961, hsa04724, hsa04727, hsa04725, hsa04728, hsa04726, hsa04720, hsa04723, hsa04740, hsa04742, hsa04750, hsa04211, hsa04213, hsa04713, hsa04714, hsa05200, hsa05205, hsa05203, hsa05012, hsa05020, hsa05030, hsa05031, hsa05032, hsa05414, hsa04934, hsa05110, hsa05166, hsa05163, hsa05169, hsa05165, hsa05146, hsa01522
PRKACG	protein kinase cAMP-activated catalytic subunit gamm	NM_002732.3	hsa04014, hsa04010, hsa04310, hsa04340, hsa04371, hsa04020, hsa04024, hsa04140, hsa04114, hsa04530, hsa04540, hsa04611, hsa04062, hsa04911, hsa04910, hsa04922, hsa04923, hsa04912, hsa04913, hsa04915, hsa04914, hsa04921, hsa04926, hsa04918, hsa04919, hsa04928, hsa04916, hsa04924, hsa04925, hsa04927, hsa04261, hsa04270, hsa04970, hsa04971, hsa04976, hsa04962, hsa04961, hsa04724, hsa04727, hsa04725, hsa04728, hsa04726, hsa04720, hsa04723, hsa04740, hsa04742, hsa04750, hsa04211, hsa04213, hsa04713, hsa04714, hsa05200, hsa05205, hsa05203, hsa05012, hsa05020, hsa05030, hsa05031, hsa05032, hsa05414, hsa04934, hsa05110, hsa05166, hsa05163, hsa05169, hsa05165, hsa05146, hsa01522
PRKAR2A			
PRKCA			

PRL			
PSMB8	proteasome subunit beta 8	NM_004159.4	hsa03050
PSMB9	proteasome subunit beta 9	NM_002800.4	hsa03050
PTEN			
PTGER4	prostaglandin E receptor 4	NM_000958.2	hsa04080, hsa04924, hsa04750, hsa05200, hsa05163, hsa05165
PTGS2	prostaglandin-endoperoxide synthase 2	NM_000963.3	hsa00590, hsa04370, hsa04064, hsa04668, hsa04625, hsa04657, hsa04923, hsa04913, hsa04921, hsa04726, hsa04723, hsa05200, hsa05206, hsa05204, hsa05222, hsa05163, hsa05167, hsa05165, hsa05140
PTH			
PTPN11			
PTPRR			
PVALB			
RAC1			
RAC2			
RANBP2			
RANBP3L			
RASGRP3	RAS guanyl releasing protein 3	NM_001139488.1	hsa04014, hsa04015, hsa04010, hsa04662, hsa05200
RB1			
REM1			
REV3L			
RGAG1	retrotransposon gag domain containing 1	NM_020769.2	
RHO			
RHOA	ras homolog family member A	NM_001664.3	hsa04014, hsa04015, hsa04310, hsa04350, hsa04072, hsa04071, hsa04024, hsa04022, hsa04150, hsa04144, hsa04510, hsa04520, hsa04530, hsa04810, hsa04611, hsa04621, hsa04625, hsa04660, hsa04670, hsa04062, hsa04921, hsa04928, hsa04270, hsa04972, hsa04722, hsa04360, hsa05200, hsa05206, hsa05205, hsa05203, hsa05210, hsa05418, hsa05130, hsa05133, hsa05152, hsa05100, hsa05163
RIPK4	receptor interacting serine/threonine kinase 4	NM_020639.2	
RIT2			
RPL27A			
RPS27A	ribosomal protein S27a	NM_002954.5	hsa03010
RSAD2	radical S-adenosyl methionine domain containing 2	NM_080657.4	
RTP4	receptor transporter protein 4	NM_022147.2	
SAMD9	sterile alpha motif domain containing 9	NM_017654.3	
SAMD9L	sterile alpha motif domain containing 9 like	NM_152703.4	
SAMHD1	SAM and HD domain containing deoxynucleoside tripl	NM_015474.3	
SCAND3			
SCIN	scinderin	NM_001112706.2	hsa04810, hsa04666, hsa05203
SCN3A	sodium voltage-gated channel alpha subunit 3	NM_006922.3	hsa04742
SELV			
SEMA3A	semaphorin 3A	NM_006080.2	hsa04360
SEMA3D	semaphorin 3D	NM_152754.2	hsa04360
SERPINB2	serpin family B member 2	NM_001143818.1	hsa04610
SETD2			
SH3GL1			
SHH			
SIN3A	SIN3 transcription regulator family member A	NM_001145358.1	hsa04919, hsa05202, hsa05016
SLC25A1			
SLC27A2			
SMAD2			
SMAD3			
SMARCA2			
SNORA2A			
SNORD67			
SNRPN			
SP1	Sp1 transcription factor	NM_138473.2	hsa04350, hsa04137, hsa04915, hsa04928, hsa04927, hsa05200, hsa05231, hsa05202, hsa05224, hsa05016, hsa04934, hsa05163, hsa01522
SP100	SP100 nuclear antigen	NM_001080391.1	hsa05203, hsa05168
SP110	SP110 nuclear body protein	NM_004509.3	
SP140L			
SPINT4			
SPRR1B			
SPTAN1	spectrin alpha, non-erythrocytic 1	NM_001130438.2	hsa04210
SRC	SRC proto-oncogene, non-receptor tyrosine kinase	NM_005417.4	hsa04015, hsa04012, hsa04370, hsa04144, hsa04137, hsa04510, hsa04520, hsa04530, hsa04540, hsa04810, hsa04611, hsa04625, hsa04062, hsa04912, hsa04915, hsa04917, hsa04921, hsa04926, hsa04919, hsa04727, hsa04750, hsa04360, hsa05205, hsa05203, hsa05219, hsa05418, hsa05120, hsa05131, hsa05152, hsa05100, hsa05161, hsa05163, hsa05167, hsa01521, hsa01522
SSRP1			
SST			
SSTR2			
STAMBPL1			
STAT1	signal transducer and activator of transcription 1	NM_007315.3	hsa04630, hsa04217, hsa04620, hsa04621, hsa04625, hsa04658, hsa04659, hsa04062, hsa04917, hsa04919, hsa04380, hsa05200, hsa05212, hsa05321, hsa04933, hsa05152, hsa05162, hsa05164, hsa05161, hsa05160, hsa05168, hsa05167, hsa05165, hsa05145, hsa05140
STAT2	signal transducer and activator of transcription 2	NM_005419.3	hsa04630, hsa04217, hsa04621, hsa04625, hsa04062, hsa04380, hsa05200, hsa05162, hsa05164, hsa05161, hsa05160, hsa05168, hsa05167, hsa05165
STAT3	signal transducer and activator of transcription 3	NM_139276.2	hsa04630, hsa04066, hsa04068, hsa04217, hsa04550, hsa04659, hsa04062, hsa04920, hsa04917, hsa05200, hsa05206, hsa05205, hsa05203, hsa05212, hsa05221, hsa05223, hsa05321, hsa04931, hsa04933, hsa05162, hsa05161, hsa05160, hsa05163, hsa05167, hsa05169, hsa05145, hsa01521
SULT4A1			
SYK			
TAF1	TATA-box binding protein associated factor 1	NM_004606.4	hsa03022
TAOK1			
TAP1	transporter 1, ATP binding cassette subfamily B memb	NM_000593.5	hsa02010, hsa04145, hsa04612, hsa05340, hsa05168, hsa05163

TAS2R13			
TAS2R8			
TCEAL7			
TDRD7	tudor domain containing 7	NM_014290.2	
TEX9			
TGFB1	transforming growth factor beta 1	NM_000660.6	hsa04010, hsa04350, hsa04390, hsa04068, hsa04060, hsa04110, hsa04218, hsa04659, hsa04672, hsa04926, hsa04380, hsa05200, hsa05205, hsa05210, hsa05212, hsa05225, hsa05226, hsa05220, hsa05211, hsa05323, hsa05321, hsa05410, hsa05414, hsa04932, hsa04933, hsa05152, hsa05166, hsa05161, hsa05146, hsa05144, hsa05145, hsa05140, hsa05142
TKTL2			
TLR3	toll like receptor 3	NM_003265.2	hsa04217, hsa04620, hsa05164, hsa05161, hsa05160, hsa05168, hsa05167, hsa05165
TLR4			
TMEM140	transmembrane protein 140	NM_018295.4	
TNF	tumor necrosis factor	NM_000594.3	hsa04010, hsa04350, hsa04064, hsa04668, hsa04071, hsa04150, hsa04060, hsa04210, hsa04217, hsa04640, hsa04620, hsa04621, hsa04622, hsa04625, hsa04650, hsa04612, hsa04660, hsa04657, hsa04664, hsa04920, hsa04380, hsa05205, hsa05310, hsa05322, hsa05323, hsa05321, hsa05330, hsa05332, hsa05010, hsa05014, hsa05418, hsa05410, hsa05414, hsa04930, hsa04940, hsa04932, hsa04931, hsa04933, hsa05133, hsa05134, hsa05152, hsa05166, hsa05164, hsa05161, hsa05160, hsa05168, hsa05163, hsa05165, hsa05146, hsa05144, hsa05145, hsa05140, hsa05142, hsa05143, hsa01523
TNFAIP3	TNF alpha induced protein 3	NM_001270508.1	hsa04064, hsa04668, hsa04217, hsa04621, hsa04657, hsa05162, hsa05169
TNFRSF10A			
TNFRSF17			
TNFSF10	TNF superfamily member 10	NM_003810.3	hsa04068, hsa04060, hsa04210, hsa04217, hsa04650, hsa05162, hsa05164
TNFSF13B	TNF superfamily member 13b	NM_006573.4	hsa04064, hsa04060, hsa04672, hsa05323
TOP2A	DNA topoisomerase II alpha	NM_001067.3	hsa01524
TOP2B	DNA topoisomerase II beta	NM_001330700.1	hsa01524
TP53	tumor protein p53	NM_001276760.1	hsa04010, hsa04310, hsa04071, hsa04151, hsa04137, hsa04110, hsa04210, hsa04216, hsa04115, hsa04218, hsa04919, hsa04722, hsa04211, hsa05200, hsa05230, hsa05202, hsa05206, hsa05205, hsa05203, hsa05210, hsa05212, hsa05225, hsa05226, hsa05214, hsa05216, hsa05220, hsa05217, hsa05218, hsa05219,hsa05215, hsa05213, hsa05224, hsa05223, hsa05014, hsa05016, hsa05418, hsa05166, hsa05162, hsa05161, hsa05160, hsa05168, hsa05163, hsa05167, hsa05169, hsa05165,hsa01524, hsa01522
TPPP2			
TRAF1	TNF receptor associated factor 1	NM_005658.4	hsa04064, hsa04668, hsa04210, hsa05200, hsa05202, hsa05203, hsa05222, hsa05168, hsa05169
TRAF6			
TRIM14			
TRIM21	tripartite motif containing 21	NM_003141.3	hsa05322
TRIM22	tripartite motif containing 22	NM_006074.4	
TRIM25			
TRIM28	tripartite motif containing 28	NM_005762.2	
34 /// TRIM6-TI	tripartite motif containing 34	NM_021616.5	
TRIM5	tripartite motif containing 5	NM_033092.2	
TSPAN19			
TUBB8			
UBA52	ubiquitin A-52 residue ribosomal protein fusion produc	NM_001033930.2	hsa03010
UBA7	ubiquitin like modifier activating enzyme 7	NM_003335.2	hsa04120, hsa05012
UBB	ubiquitin B	NM_018955.3	hsa04144, hsa04137, hsa05012, hsa05167
UBC	ubiquitin C	NM_021009.6	hsa03320
UBE2L6	ubiquitin conjugating enzyme E2 L6	NM_004223.4	hsa04120, hsa05012
UBL4B			
UCKL1			
UHMK1			
UMPS	uridine monophosphate synthetase	NM_000373.3	hsa00240, hsa00983
USP18	ubiquitin specific peptidase 18	NM_017414.3	
USP50			
VCX3A			
VEGFA	vascular endothelial growth factor A	NM_001171623.1	hsa04014, hsa04015, hsa04010, hsa04370, hsa04066, hsa04151, hsa04510, hsa04926, hsa05200, hsa05206, hsa05205, hsa05212, hsa05211, hsa05219, hsa05323, hsa05418, hsa04933, hsa05163, hsa05167, hsa05165, hsa01521
VPREB3			
WDFY2			
WDTC1			
WFDC5			
XAF1	XIAP associated factor 1	NM_017523.4	
YARS			
ZAP70			
ZBTB20			
ZC3HAV1	zinc finger CCCH-type containing, antiviral 1	NM_020119.3	
ZDHHC17			
ZFPM2			
ZFR			
ZNF442	zinc finger protein 442	NM_030824.2	
ZNF557			
ZNF620			
ZNF678			
ZNF79			
ZNF804A			
