# Supplementary material for: Drug Repurposing for Japanese Encephalitis Virus Infection by Systems Biology Methods
Source: Molecules. 2018 Dec 18;23(12):3346. doi: 10.3390/molecules23123346 (PMC6320907; doi:10.3390/molecules23123346)
Supplement: Supplementary file 1 [file molecules-23-03346-s001.zip › supple/table S2.pdf]

Supplementary Table 2: The potential anti-JEV agents discovered by GeneRank algorithm

Agents	Evidence in JEV treatment	Indications
ACETYLSALICYLIC ACID	Y	the pain and inflammation, thrombosis
ADALIMUMAB	N	rheumatoid arthritis, ankylosing spondylitis, psoriatic arthritis
AMSACRINE	N	acute myeloid leukaemia, lymphoma
ANDROGRAPHOLIDE	N	ulcerative colitis
APREMILAST	N	psoriasis and psoriatic disorders
APRINDINE	N	antiarrhythmic
ARSENIC TRIOXIDE	N	acute promyelocytic leukemia (APL)
AV411	N	neuropathic pain
BAY80-6946 (COPANLISIB)	N	relapsed follicular lymphoma (FL)
BEPRIDIL	N	hypertension, chronic stable angina, antiarrhythmic
BEVACIZUMAB	N	metastatic colorectal cancer, HER2-negative metastatic breast cancer
BGJ398	N	bladder cancer, cholangiocarcinoma
BINIMETINIB	N	metastatic melanoma
BORTEZOMIB	N	multiple myeloma, lymphoma
BUPARLISIB HYDROCHLORIDE	N	breast cancer
BYL719 (ALPELISIB)	N	breast cancer
CAFFEINE	N	fatigue, neurasthenia
CARBAMAZEPINE	N	epilepsy
CELECOXIB	N	osteoarthritis (OA), rheumatoid arthritis (RA), juvenile rheumatoid arthritis (JRA), ankylosing spondylitis,
CETUXIMAB	N	metastatic colorectal carcinoma,
CH5132799	N	cancer
CHLORPROMAZINE	Y	schizophrenia, intermittent porphyria
CINCHOCAINE	N	anesthesia
CRX-139	N	rheumatoid arthritis
CUDC-101	N	breast cancer
CURCUMIN	Y	cancer, rheumatoid arthritis, inflammatory bowel disease
DACTOLISIB	N	follicular lymphoma (FL), bresat cancer, prostatic carcinoma, leukaemia
DASATINIB	N	chronic myeloid leukemia
DEXIBUPROFEN	N	analgesic, anti-inflammatory, antipyretic
DICLOFENAC	N	osteoarthritis, rheumatoid arthritis
DILMAPIMOD	N	inflammation, pain, neuropathic, arthritis, rheumatoid, coronary heart disease
DOXORUBICIN	N	acute lymphoblastic leukemia, acute myeloblastic leukemia, Wilms' tumor, neuroblastoma, soft tissue and bone sarcomas, breast carcinoma, ovarian carcinoma, transitional cell bladder carcinoma, thyroid carcinoma, gastric carcinoma
DS-7423	N	cancer
DUVELISIB	N	chronic lymphocytic leukemia (CLL), small lymphocytic lymphoma (SLL)
ENMD-2076	N	breast cancer, leukemia (unspecified), lung cancer, solid tumors
ERLOTINIB	N	non-small cell lung cancer, metastatic pancreatic cancer
ETANERCEPT	Y	rheumatoid arthritis, psoriatic arthritis, ankylosing spondylitis
EVEROLIMUS	N	breast cancer, neuroendocrine tumor,
FELODIPINE	N	hypertension
FLUNARIZINE	N	migraine, occlusive peripheral vascular disease, vertigo of central, peripheral origin
FLUPHENAZINE	N	psychotic disorders
GDC-0941	N	solid cancers, bresat cancer
GDC-0980	N	renal cell carcinoma, endometrial carcinoma, Non-Hodgkin's lymphoma
GEFITINIB	N	non-small cell lung cancer
GENISTEIN	Y	prostate cancer
GLUCOSAMINE	N	osteoarthritis
GSK2126458	N	cancer
GSK2636771	N	melanoma
HMPL-004	N	inflammatory colitis
IBUPROFEN	N	analgesic, anti-inflammatory, antipyretic
ISOFLURANE	N	general anesthesia
JAK3 INHIBITOR II	N	rheumatoid arthritis
LENALIDOMIDE	N	multiple myeloma, transfusion-dependent anemia
LOPERAMIDE	N	nonspecific diarrhea, chronic diarrhea
LY 294002	N	colon cancer
MELATONIN	N	sleep disorders
MINOCYCLINE	Y	infections caused by susceptible strains of microorganisms
MOMELOTINIB	N	myelofibrosis
NICARDIPINE	N	angina, hypertension
NIFEDIPINE	N	vasospastic angina, chronic stable angina, hypertension, and Raynaud's phenomenon
OXALIPLATIN	N	colon carcinoma, rectum carcinoma
PACLITAXEL	N	Kaposi's sarcoma, lung cancer, ovarian cance, breast cancer
PANITUMUMAB	N	colorectal carcinoma
PERPHENAZINE	N	psychotic disorders
PF-4691502	N	breast cancer
PHENOXYBENZAMINE	N	phaeochromocytoma (malignant), benign prostatic hypertrophy, malignant essential hypertension
PIMOZIDE	N	tourette's disorder
PKI-58 (Gedatolisib)	N	breast cancer, lung cancer squamous cell
PRENYLAMINE	N	angina pectoris, myocardial infarction, coronary atherosclerosis
PROMETHAZINE	N	allergic disorders, nausea/vomiting
PSEUDOEPHEDRINE	N	nasal congestion, sinus congestion, eustachian tube congestion, vasomotor rhinitis, allergic rhinitis, croup, sinusitis, otitis media, tracheobronchitis
PWT33597	N	malignancies
PX-866	N	prostate cancer
RESVERATROL	N	Herpes labialis infections

RIBAVIRIN	Y	Hepatitis C virus (HCV) infection
RIGOSERTIB	N	chronic myelomonocytic leukemia
SF1126	N	cancer
SOPHORETIN (QUERCETIN)	N	cough, hypertension
SORAFENIB	N	hepatocellular carcinoma, renal cell carcinoma
TALMAPIMOD	N	rheumatoid arthritis
TEMSIROLIMUS	N	renal cell carcinoma (RCC), breast cancer, lymphoma, rheumatoid arthritis, multiple myeloma
TG-100-115	N	angioedema, myocardial infarction
THALIDOMIDE	N	erythema nodosum leprosum (ENL)
TRASTUZUMAB	N	breast cancer
TRIFLUOPERAZINE	N	anxiety disorders, depressive symptoms
VALPROIC ACID	Y	seizure disorders, mania, migraine headache
VANDETANIB	N	thyroid cancer
VX-702	N	coronary artery disease, inflammatory disorders, rheumatoid arthritis
XL147	N	endometrial cancer, endometrial neoplasms
XL765	N	cancer
ZSTK474	N	cancer
