# Supplementary material for: Drug Repurposing for Japanese Encephalitis Virus Infection by Systems Biology Methods
Source: Molecules. 2018 Dec 18;23(12):3346. doi: 10.3390/molecules23123346 (PMC6320907; doi:10.3390/molecules23123346)
Supplement: Supplementary file 1 [file molecules-23-03346-s001.zip › supple/table S3.pdf]

Supplementary Table 3: The significant sub-networks associated with encephalitis

Subnetwork index	Components					
1	ATM	BIRC7	NOL7	NRBP1	PHF1	PKNOX1	STK11
2	ACACA	ARHGAP1	LARS	PC	PSMD10		
3	ADAR	FOXG1	IFI16	PSMA7	WDR46		
4	LMO4	SEC23IP	SLAMF1	TPM3	VPS52		
5	PSMB8	PSMB9	TAP1	TAP2	TAPBP		
6	BRD2	BRD7	RPLP1	RPS18			
7	DLEU1	QKI	SETDB1	TXNDC9			
8	HAX1	NDUFS2	NTM	SDHC			
9	HLA-DMA	HLA-DMB	HLA-DOA	HLA-DRB1			
10	ANKS1A	ARHGAP10	PAK2				
11	ASAH2	KIFC1	PARD6B				
12	B4GALT3	HLA-DPA1	HLA-DPB1				
13	CUTA	PPP4R2	SMEK2				
14	EEF1A2	FRAS1	NXPH2				
15	FOXP4	NRAS	RGL2				
16	HLA-DQA1	HLA-DQA2	HLA-DQB1				
